# A practical and efficient approach to imidazo[1,2-*a*]pyridine-fused isoquinolines through the post-GBB transformation strategy

**DOI:** 10.3762/bjoc.13.82

**Published:** 2017-05-04

**Authors:** Taofeng Shao, Zhiming Gong, Tianyi Su, Wei Hao, Chao Che

**Affiliations:** 1 Laboratory of Chemical Genomics, Engineering Laboratory for Chiral Drug Synthesis, School of Chemical Biology and Biotechnology, Peking University Shenzhen Graduate School, Shenzhen 518055, China

**Keywords:** Groebke–Blackburn–Bienaymé reaction, imidazo[1,2*-a*]pyridines, isoquinolines, multicomponent reaction, Ugi reaction

## Abstract

Diversity-oriented synthesis of the biologically intriguing imidazo[1,2-*a*]pyridine-fused isoquinoline systems from readily available starting materials was achieved through the Groebke–Blackburn–Bienaymé reaction followed by a gold-catalyzed cyclization strategy. The synthetic approach is characterized by mild reaction conditions and a broad substrate scope, allowing for the rapid construction of structurally complex and diverse heterocycles in moderate to good yields.

## Introduction

**Figure 1:**
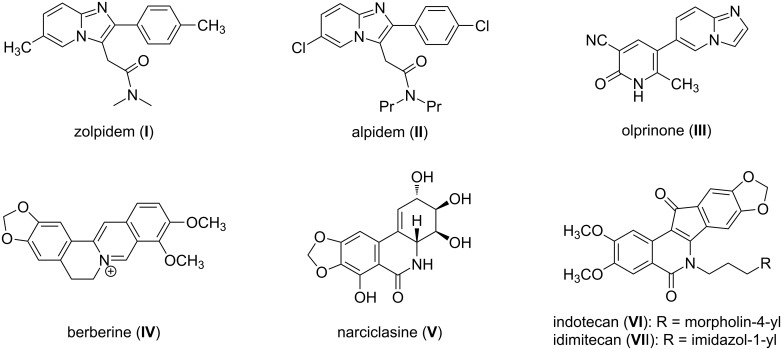
Representative bioactive imidazo[1,2-*a*]pyridine and isoquinoline-containing derivatives.

Imidazo[1,2-*a*]pyridines have been reported to display a wide range of biological activities [1–5], and these skeletons are found in various clinical drugs such as zolpidem (**I**), alpidem (**II**), and olprinone (**III**), which were approved for the treatment of insomnia, anxiety and acute heart failure, respectively (Figure 1) [6]. Furthermore, the isoquinoline motif represents a privileged medicinal skeleton widely found in a number of natural alkaloids and pharmaceutically active compounds [7]. Some of them exhibit diversified biological properties, including anti-inflammatory [8], antibacterial [9], antiviral [10], and antitumor activities [11]. For example, the natural alkaloids berberine (**IV)** and narciclasine (**V**) possess antiplasmodial and antiviral activity, respectively [12–13]. Indotecan (**VI**) and its analog idimitecan (**VII**) were identified as topoisomerase I inhibitors, and were promoted into phase I clinical trials [14].

Multicomponent reactions (MCRs) [15–19], comprising three or more components, provide straightforward approaches to a wide range of heterocycles through the formation of various bonds in a one-pot process. These reactions not only greatly accelerate chemical syntheses [20], but also allow access to diverse chemical structures [21] from readily accessible building blocks. In the past decades, considerable efforts have been made towards the development of new MCRs and their application to the diversity-oriented synthesis of biologically relevant molecules for drug discovery [22–27].

The Ugi reaction [28], an elegant pioneer of a multicomponent reaction, represents a powerful synthetic tool to assemble versatile peptide-like compounds. It has found many applications in the facile synthesis of natural products and biologically interesting molecules [29–30]. Although the Ugi-4CR generates linear α-acylamino-amides, a wide range of heterocycles are accessible through the combination with other transformations (post-transformation strategy) [31]. For example, the Ugi/Diels–Alder process leads to the formation of benzofurans and indoles [32] as well as to structurally complex polycyclic ring systems [33]; an Ugi/aza-Wittig process allowed for the synthesis of 2,5-disubstituted 1,3,4-oxadiazoles [34]; the Ugi/Pictet–Spengler sequence provided a rapid and efficient approach to polycyclic natural product-like alkaloids [35]. Accordingly, the combination of the Ugi reaction with other transformations proved to be powerful strategies for the efficient synthesis of novel heterocycles. In 1998, the Groebke–Blackburn–Bienaymé (GBB) reaction, an Ugi-3CR variant was discovered by three groups independently [36–38]. The GBB reaction of an amidine, an aldehyde and an isocyanide proceeds through the isocyanide-involving formal [4 + 1] cycloaddition [39] affording the biologically important imidazo[1,2-*a*]pyridine scaffold. Due to the atom and step economy, high efficiency and intriguing biological profiles of the products, the GBB reaction has attracted broad attention in the field of organic synthesis [40–42]. In order to expand the structural diversity of GBB products, further investigation of GBB-based synthetic strategies remains highly desirable.

In continuation of our research on the development of MCR strategies for the rapid library synthesis of biologically interesting heterocycles [43–47], we were interested in a practical synthetic strategy towards imidazo[1,2-*a*]pyridine-fused isoquinoline systems. We believe that this type of polycyclic systems may have interesting biological profiles [48]. Herein, we report our recent efforts on the development of a post-GBB transformation strategy for the concise synthesis of diverse imidazo[1,2-*a*]pyridine-fused isoquinoline systems.

## Results and Discussion

**Scheme 1:**
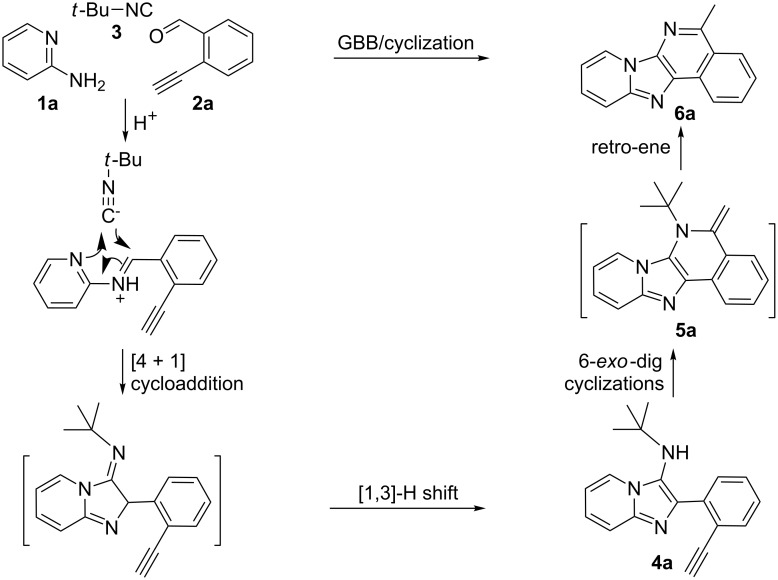
GBB-based MCR strategy for the imidazo[1,2-*a*]pyridine-fused isoquinoline derivatives.

From a design perspective, we envisioned that the imidazo[1,2-*a*]pyridine-fused isoquinoline **6a** [49–50] could be constructed through a GBB reaction/cyclization strategy (Scheme 1). The intermediate GBB product **4a** could be constructed starting from 2-ethynylbenzaldehyde (**2a**) through an imine formation/formal [4 + 1] cycloaddition/[1,3]-H shift. The so obtained GBB product imidazo[1,2-*a*]pyridine **4a** bearing an amino group and an acetylene unit may then undergo a sequential 6-*exo*-dig cyclization/retro-ene reaction to form the desired imidazo[1,2-*a*]pyridine-fused isoquinoline **6a**. The cyclization reaction could be realized with the aid of silver or gold catalysts [51–52].

With this idea in mind, we commenced our studies by investigating the GBB reaction of 2-aminopyridine (**1a**), 2-ethynylbenzaldehyde (**2a**) and *tert*-butylisocyanide (**3**). The GBB reaction proceeded smoothly in MeOH in the presence of catalytic PTSA or HClO_4_ at room temperature to afford imidazo[1,2-*a*]pyridine **4a** in 90% yield, and the cyclized product **6a** was not detected under these mild conditions. Subsequent heating of **4a** in refluxing 1,4-dioxane or toluene failed to deliver the expected product **6a**, even under acidic or basic conditions.

Then, we turned to Ag and Au catalysts and investigated the metal-catalyzed intramolecular cyclization reaction of **4a** and the results are collected in Table 1. First, we investigated AgOTf as the catalyst, which afforded the cyclized product **6a** in 12% yield in refluxing CH_2_Cl_2_ in the presence of 10 mol % of catalyst. The yield was increased to 45% when replacing CH_2_Cl_2_ with CHCl_3_, whereas only a trace amount of the desired product was obtained in refluxing CH_3_CN or 1,4-dioxane (Table 1, entries 1–4). It revealed that the solvent plays a key role in this cyclization reaction. For comparison, we tested also AgSbF_6_ as the catalyst and it was found to be less effective than AgOTf (Table 1, entry 5). To improve the reaction efficiency, we next evaluated the cyclization reaction in refluxing CHCl_3_ in the presence of a range of Au catalysts. Although almost no reaction took place with Au(PPh_3_)Cl as the catalyst, the use of Au(PPh_3_)NTf_2_ resulted in a satisfactory yield (70%) of the product (Table 1, entries 6–9). Motivated by this result, other Au catalysts were further surveyed, and Au(JohnPhos)Cl was found to be the most efficient, delivering **6a** in 78% yield (Table 1, entries 10–14). Next, the effect of the solvent on the reaction was tested and replacement of CHCl_3_ with CH_3_CN led to a slightly enhanced yield (83%) (Table 1, entries 15 and 16). Additionally, in refluxing CH_3_CN no other Au catalysts afforded better results than Au(JohnPhos)Cl (Table 1, entries 17 and 18). Overall, the optimal conditions for the cyclization reaction are as follows: Au(JohnPhos)Cl (10 mol %), CH_3_CN, reflux, 24 h.

**Table 1:** Optimization of the cyclization reaction conditions.^a^

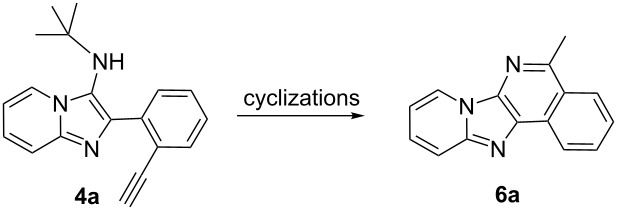			

Entry	Catalyst	Solvent	Yield^b^ (%)

1	AgOTf	CH_2_Cl_2_	12
2	AgOTf	CHCl_3_	45
3	AgOTf	CH_3_CN	trace
4	AgOTf	dioxane	trace
5	AgSbF_6_	CHCl_3_	38
6	Au(PPh_3_)Cl	CHCl_3_	trace
7	Au(PPh_3_)OTf	CHCl_3_	42
8	Au(PPh_3_)SbF_6_	CHCl_3_	21
9	Au(PPh_3_)NTf_2_	CHCl_3_	70
10	Au_2_(dppe) (SbF_6_)_2_	CHCl_3_	51
11	Au_2_(binap)( SbF_6_)_2_	CHCl_3_	53
12	Au(JohnPhos)Cl	CHCl_3_	78
13	Au(JohnPhos)OTf	CHCl_3_	42
14	Au(JohnPhos)SbF_6_	CHCl_3_	74
**15**	**Au(JohnPhos)Cl**	**CH_3_CN**	**83**
16	Au(JohnPhos)Cl	dioxane	49
17	Au_2_(dppe) Cl_2_	CH_3_CN	34
18	Au_2_(binap)Cl_2_	CH_3_CN	42

^a^General conditions: substrate **4a** (0.2 mmol), catalyst (10 mol %), solvent (2 mL) at reflux temperature for 24 h. ^b^Isolated yield.

With the optimal conditions at hand, we then set out to explore the reaction scope for the library generation of structurally diverse imidazo[1,2-*a*]pyridine-fused isoquinolines and the results are collected in Table 2. Initially, several GBB adducts **4** were synthesized through GBB reaction of amidines **1**, substituted 2-ethynylbenzaldehydes **2** and *tert*-butylisocyanide (**3**). Indeed, the acetylene group in the aldehyde component had no obvious steric effect on the efficiency of the GBB reaction affording the GBB product in good to excellent yields in most cases. On the other hand, the substituent ortho to the amino group in the amidine component had a negative effect on the GBB reaction efficiency due to steric hindrance (Table 2, entries 6, 11, 13 and 17).

**Table 2:** Substrate scope for the syntheses of compounds **4** and **6**.^a^

Entry	Starting materials	GBB product **4**	Yield^b^ (%)	Cyclized product **6**	Yield^b^ (%)

1	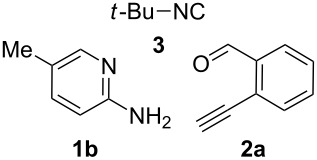	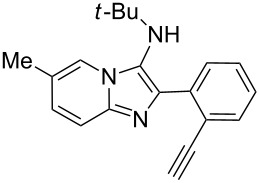 **4b**	94	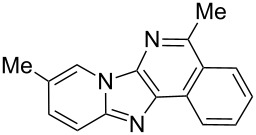 **6b**	72
2	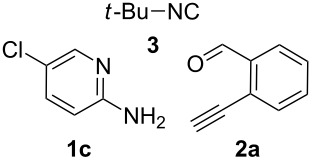	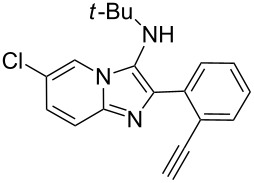 **4c**	80	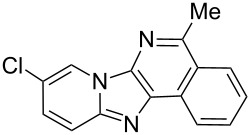 **6c**	75
3	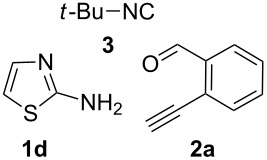	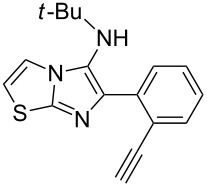 **4d**	62	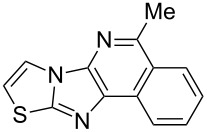 **6d**	61
4	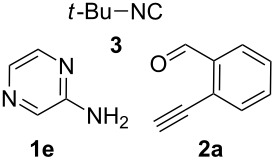	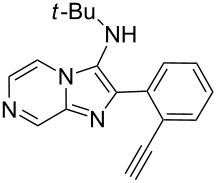 **4e**	96	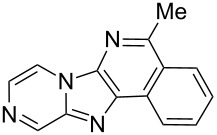 **6e**	63
5	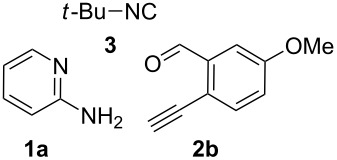	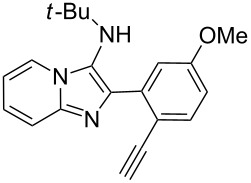 **4f**	89	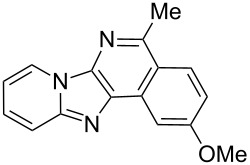 **6f**	78
6	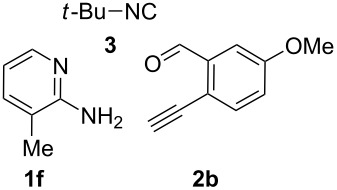	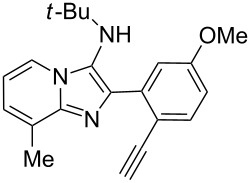 **4g**	61	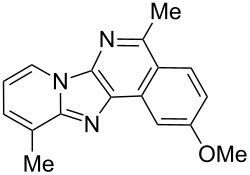 **6g**	80
7	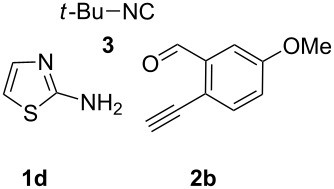	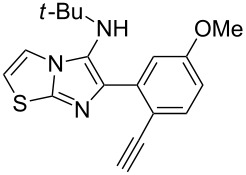 **4h**	85	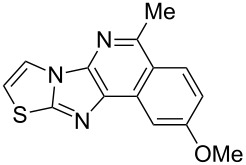 **6h**	56
8	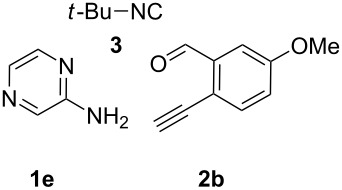	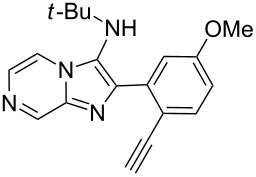 **4i**	88	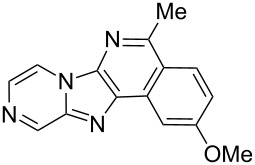 **6i**	58
9	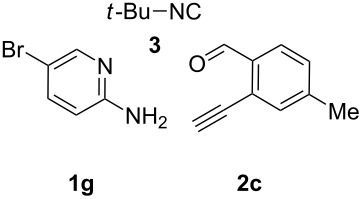	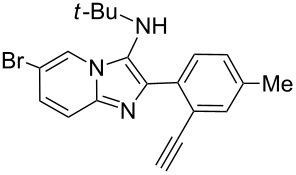 **4j**	64	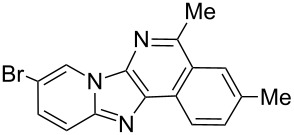 **6j**	78
10	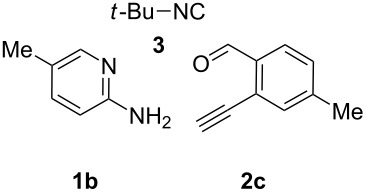	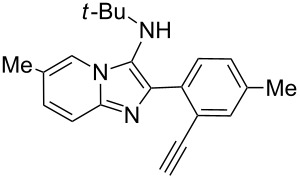 **4k**	75	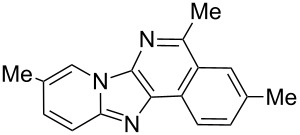 **6k**	87
11	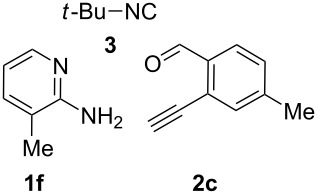	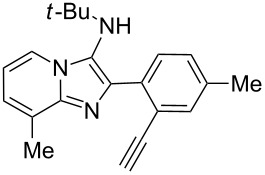 **4l**	49	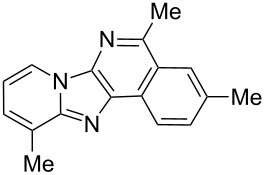 **6l**	79
12	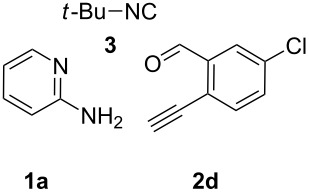	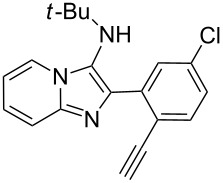 **4m**	71	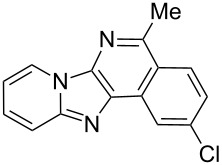 **6m**	48
13	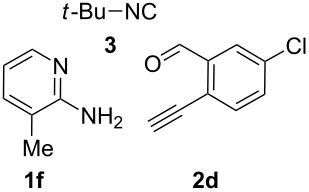	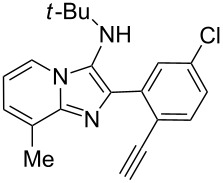 **4n**	47	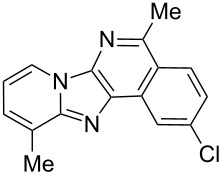 **6n**	55
14	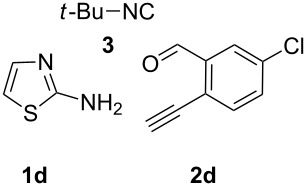	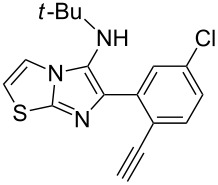 **4o**	54	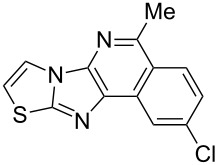 **6o**	62
15	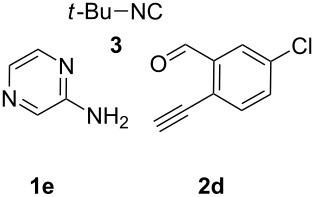	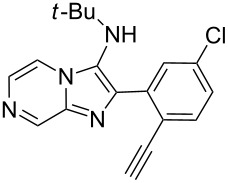 **4p**	95	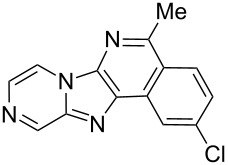 **6p**	63
16	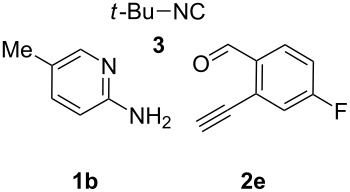	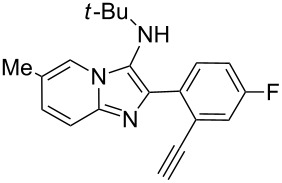 **4q**	74	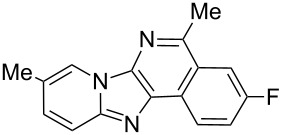 **6q**	58
17	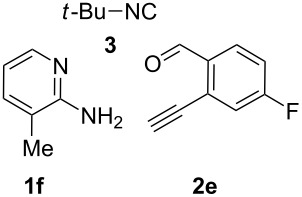	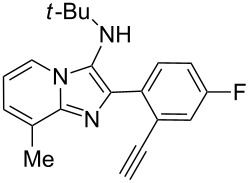 **4r**	43	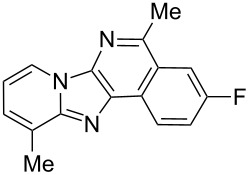 **6r**	67
18	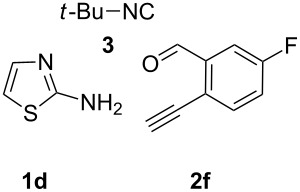	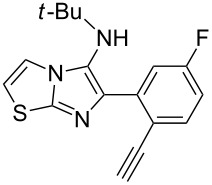 **4s**	57	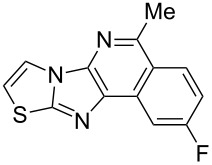 **6s**	59

^a^GBB reaction conditions: **1** (0.5 mmol), **2** (0.5 mmol), **3** (0.6 mmol), MeOH (1 mL); PTSA (5%), room temperature, **12h**; annulation conditions: substrate **4** (0.2 mmol), Au(JohnPhos)Cl (10 mol %), CH_3_CN (2 mL) at reflux temperature for 24 h. ^b^Isolated yields.

Then, the newly generated GBB adducts **4b**–**s** were exposed to the established cyclization conditions to deliver the corresponding imidazo[1,2-*a*]pyridine-fused isoquinolines **6b**–**s** in moderate to good yields, and their structures were unambiguously confirmed by ^1^H NMR, ^13^C NMR, and HRMS analysis. Various functionalities related to the amidine and aldehyde components, including electron-donating methoxy and methyl groups or electron-withdrawing halides, were well tolerated. Generally, the substitution pattern of the amidine moiety had little effect on the Au-catalyzed annulation reaction, whereas neutral or electron-donating groups on the aldehyde moiety gave a higher yield in comparison with the electron-withdrawing halides. Notably, bromo-substituted substrates were also tolerated the reaction conditions, allowing for the further manipulation through various cross-coupling reaction (Table 2, entry 9).

## Conclusion

In conclusion, we have developed a practical and efficient synthetic approach to structurally diverse imidazo[1,2-*a*]pyridine-fused isoquinolines with moderate to good yields through the GBB multicomponent reaction/Au-catalyzed cyclization strategy. The described method provides a new tool for a rapid compound library generation from readily accessible starting materials. Further, the protocol tolerates a broad substrate scope, which will make it attractive for the application in parallel synthesis and combinatorial chemistry.

## Experimental

**Typical procedure for the GBB multicomponent reaction.** To a solution of 2-aminopyridine (**1a**, 0.5 mmol), 2-ethynylbenzaldehyde (**2a**, 0.5 mmol), and *tert*-butylisocyanide (**3**, 0.6 mmol) in 1 mL of methanol were added *p*-toluenesulfonic acid (4.7 mg, 0.025 mmol) and the reaction mixture was stirred at rt for 12 h. The mixture was diluted with 15 mL of dichloromethane and washed successively with water (10 mL), saturated NaHCO_3_ solution (10 mL) and brine (10 mL). After drying over anhydrous Na_2_SO_4_, the mixture was concentrated under vacuum and the resulting residue was purified by flash chromatography (hexane/ethyl acetate 8:1) to afford GBB adduct **4a** (90% yield).

**Typical procedure for the Au-catalyzed cyclization reaction.** To a solution of the GBB adduct **4** (0.2 mmol) in 2 mL of acetonitrile was added Au(JohnPhos)Cl (0.02 mmol) and the resulting mixture was stirred under inert atmosphere at reflux temperature for 24 h. Then, the solvent was removed under vacuum and the residue purified by flash chromatography (hexane/ethyl acetate 5:1) to afford the desired product **6**.

## Supporting Information

File 1 Characterization data for all compounds and copies of NMR spectra for compounds **6a**–**s**.

## References

[1] Tantry S J, Markad S D, Shinde V, Bhat J, Balakrishnan G, Gupta A K, Ambady A, Raichurkar A, Kedari C, Sharma S (2017). J Med Chem.

[2] Shukla N M, Salunke D B, Yoo E, Mutz C A, Balakrishna R, David S A (2012). Bioorg Med Chem.

[3] Hamdouchi C, de Blas J, del Prado M, Gruber J, Heinz B A, Vance L (1999). J Med Chem.

[4] Frett B, McConnell N, Smith C C, Wang Y, Shah N P, Li H-y (2015). Eur J Med Chem.

[5] Bode M L, Gravestock D, Moleele S S, van der Westhuyzen C W, Pelly S C, Steenkamp P A, Hoppe H C, Khan T, Nkabinde L A (2011). Bioorg Med Chem.

[6] Enguehard-Gueiffier C, Gueiffier A (2007). Mini-Rev Med Chem.

[7] Bentley K W (1998). Nat Prod Rep.

[8] Charpiot B, Bitsch F, Buchheit K-H, Channez P, Mazzoni L, Mueller T, Vachier I, Naef R (2001). Bioorg Med Chem.

[9] Kim S-H, Shin D-S, Oh M-N, Chung S-C, Lee J-S, Oh K-B (2004). Biosci, Biotechnol, Biochem.

[10] Kartsev V G (2004). Med Chem Res.

[11] Chaniyara R, Kapuriya N, Dong H, Lee P-C, Suman S, Marvania B, Chou T-C, Lee T-C, Kakadiya R, Shah A (2011). Bioorg Med Chem.

[12] Wright C W, Marshall S J, Russell P F, Anderson M M, Phillipson J D, Kirby G C, Warhurst D C, Schiff P L (2000). J Nat Prod.

[13] Gabrielsen B, Monath T P, Huggins J W, Kefauver D F, Pettit G R, Groszek G, Hollingshead M, Kirsi J J, Shannon W M, Schubert E M (1992). J Nat Prod.

[14] Peterson K E, Cinelli M A, Morrell A E, Mehta A, Dexheimer T S, Agama K, Antony S, Pommier Y, Cushman M (2011). J Med Chem.

[15] Dömling A, Ugi I (2000). Angew Chem, Int Ed.

[16] Dömling A (2006). Chem Rev.

[17] Zhu J, Bienaymé H (2005). Multicomponent Reactions.

[18] Koopmanschap G, Ruijter E, Orru R V A (2014). Beilstein J Org Chem.

[19] Rotstein B H, Zaretsky S, Rai V, Yudin A K (2014). Chem Rev.

[20] Bienaymé H, Hulme C, Oddon G, Schmitt P (2000). Chem – Eur J.

[21] Wess J, Urmann M, Sickenberger B (2001). Angew Chem, Int Ed.

[22] Koszytkowska-Stawińska M, Buchowicz W (2014). Beilstein J Org Chem.

[23] Haji M (2016). Beilstein J Org Chem.

[24] Hulme C, Ayaz M, Martinez-Ariza G, Medda F, Shaw A, Czechtizky W, Hamley P (2015). Recent Advances in Multicomponent Reaction Chemistry: Applications in Small Molecule Drug Discovery. Small Molecule Medicinal Chemistry: Strategies and Technologies.

[25] Dömling A, Wang W, Wang K (2012). Chem Rev.

[26] Akritopoulou-Zanze I (2008). Curr Opin Chem Biol.

[27] Weber L (2002). Curr Med Chem.

[28] Ugi I, Steinbrückner C (1960). Angew Chem.

[29] Touré B B, Hall D G (2009). Chem Rev.

[30] Bauer S M, Armstrong R W (1999). J Am Chem Soc.

[31] Zhu J (2003). Eur J Org Chem.

[32] Lu K, Luo T, Xiang Z, You Z, Fathi R, Chen J, Yang Z (2005). J Comb Chem.

[33] Lee D, Sello J K, Schreiber S L (2000). Org Lett.

[34] Ramazani A, Rezaei A (2010). Org Lett.

[35] Wang W, Ollio S, Herdtweck E, Dömling A (2011). J Org Chem.

[36] Groebke K, Weber L, Mehlin F (1998). Synlett.

[37] Blackburn C, Cuan B, Fleming P, Shiosaki K, Tsai S (1998). Tetrahedron Lett.

[38] Bienaymé H, Bouzid K (1998). Angew Chem, Int Ed.

[39] Kruithof A, Ruijter E, Orru R V A (2015). Chem – Asian J.

[40] Hulme C, Lee Y-S (2008). Mol Diversity.

[41] Devi N, Rawal R K, Singh V (2015). Tetrahedron.

[42] Shaaban B, Abdel-Wahab B F (2016). Mol Diversity.

[43] Che C, Xiang J, Wang G-X, Fathi R, Quan J-M, Yang Z (2007). J Comb Chem.

[44] Che C, Li S, Jiang X, Quan J, Lin S, Yang Z (2010). Org Lett.

[45] Che C, Li S, Yu Z, Li F, Xin S, Zhou L, Lin S, Yang Z (2013). ACS Comb Sci.

[46] Che C, Yang B, Jiang X, Shao T, Yu Z, Tao C, Li S, Lin S (2014). J Org Chem.

[47] Yang B, Tao C, Shao T, Gong J, Che C (2016). Beilstein J Org Chem.

[48] Xiang J, Yang H, Che C, Zou H, Yang H, Wei Y, Quan J, Zhang H, Yang Z, Lin S (2009). PLoS One.

[49] Guchhait S K, Chaudhary V, Madaan C (2012). Org Biomol Chem.

[50] Chavignon O, Raihane M, Deplat P, Chabard J L, Gueiffier A, Blache Y, Dauphin G, Teulade J C (1995). Heterocycles.

[51] Miaskiewicz S, Gaillard B, Kern N, Weibel J-M, Pale P, Blanc A (2016). Angew Chem, Int Ed.

[52] Mirabdolbaghi R, Dudding T (2015). Org Lett.

