# Supplementary material for: A practical and efficient approach to imidazo[1,2-a]pyridine-fused isoquinolines through the post-GBB transformation strategy
Source: Beilstein J Org Chem. 2017 May 4;13:817–24. doi: 10.3762/bjoc.13.82 (PMC5433183; doi:10.3762/bjoc.13.82)
Supplement: File 1 — Characterization data for all compounds and copies of NMR spectra for compounds 6a–s. [file Beilstein_J_Org_Chem-13-817-s001.pdf]

Supporting Information

for

A practical and efficient approach to imidazo[1,2-*a*]pyridine-fused isoquinolines through the post-GBB transformation strategy

Taofeng Shao, Zhiming Gong, Tianyi Su, Wei Hao, and Chao Che*

Address: Laboratory of Chemical Genomics, Engineering Laboratory for Chiral Drug Synthesis, School of Chemical Biology and Biotechnology, Peking University Shenzhen Graduate School, Shenzhen 518055, China

Email: Chao Che - chec@pkusz.edu.cn

*Corresponding author

Characterization data for all compounds and copies of NMR spectra for compounds 6a–s

General Information

Unless noted otherwise, all reactions are performed under a nitrogen atmosphere and materials obtained from commercial suppliers were used without further purification. ^{1}H NMR spectra were recorded on a 300 MHz, 400 MHz spectrometer using residual solvent (δ ($CDCl_3$) = 7.26) as internal standard. All coupling constants are reported in hertz (Hz). ^{13}C NMR spectra were recorded on the same instruments and chemical shifts were measured relative to solvent resonances (δ ($CDCl_3$) = 77.0 ppm). High resolution mass spectra were obtained on a quadrupole time-of-flight (QqTOF) mass spectrometer utilizing electrospray ionization (ESI) method.

Characterization data

N-(*tert*-Butyl)-2-(2-ethynylphenyl)imidazo[1,2-*a*]pyridin-3-amine (**4a**)

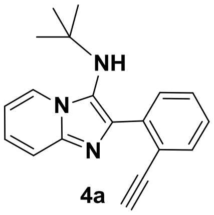

4a

Yield: 90%; ^{1}H NMR (400 MHz, $CDCl_3$) *δ* 8.31 (*d*, *J* = 6.8 Hz, 1H), 7.82 (*d*, *J* = 8.0 Hz, 1H), 7.45-7.59 (*m*, 3 H), 7.30-7.34 (*m*, 1H), 7.13 (*t*, *J* = 7.6 Hz, 1H), 6.76 (*t*, *J* = 6.8 Hz, 1H), 3.65 (*s*, 1H), 3.18 (*s*, 1H), 0.90 (*s*, 9H); ^{13}C NMR (100 MHz, $CDCl_3$) *δ* 142.4, 138.6, 138.4, 133.3, 131.1, 129.4, 127.4, 125.3, 124.1, 123.8, 120.1, 117.3, 111.2, 84.4, 79.6, 55.9, 29.8.

N-(*tert*-Butyl)-2-(2-ethynylphenyl)-6-methylimidazo[1,2-*a*]pyridin-3-amine (**4b**)

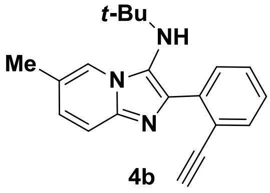

4b

Yield: 94%; ^{1}H NMR (400 MHz, $CDCl_3$) *δ* 8.09 (*s*, 1H), 7.80 (*d*, *J* = 7.2 Hz, 1H), 7.57 (*dd*, *J* = 0.8, 7.6 Hz, 1H), 7.42-7.48 (*m*, 2H), 7.29-7.33 (*m*, 1H), 6.99 (*dd*, *J* = 1.6, 9.2 Hz, 1H), 3.62 (*s*, 1H), 3.17 (*s*, 1H), 2.33 (*s*, 3H), 0.90 (*s*,

9H); ^{13}C NMR (100 MHz, $CDCl_3$) δ 141.4, 138.7, 138.3, 133.2, 131.0, 129.4, 127.4, 127.3, 125.0, 121.3, 120.7, 120.0, 116.6, 84.4, 79.5, 55.9, 29.8, 18.4.

N-(*tert*-Butyl)-6-chloro-2-(2-ethynylphenyl)imidazo[1,2-*a*]pyridin-3-amine (**4c**)

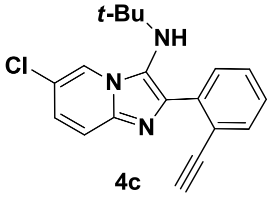

4c

Yield: 80%; ^{1}H NMR (300 MHz, $CDCl_3$): δ 8.35 (*d*, *J* = 1.5, 1H), 7.81 (*dd*, *J* = 0.9, 7.8 Hz, 1H), 7.60 (*d*, *J* = 7.8 Hz, 1H), 7.48-7.51 (*m*, 2H), 7.32-7.37 (*m*, 1H), 7.11 (*dd*, *J* = 2.1, 9.6 Hz, 1H), 3.66 (*s*, 1H), 3.19 (*s*, 1H), 0.91 (*s*, 9H); ^{13}C NMR (75 MHz, $CDCl_3$) δ 140.4, 139.5, 138.0, 133.3, 130.9, 129.4, 127.6, 125.7, 125.5, 121.6, 120.0, 119.8, 117.8, 84.2, 79.8, 55.9, 29.7.

N-(*tert*-Butyl)-6-(2-ethynylphenyl)imidazo[2,1-*b*]thiazol-5-amine (**4d**)

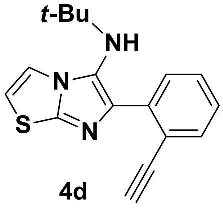

4d

Yield: 62%; ^{1}H NMR (300 MHz, $CDCl_3$) δ 7.73 (*d*, *J* = 7.8 Hz, 1H), 7.56 (*d*, *J* = 7.8 Hz, 1H), 7.41-7.46 (*m*, 2H), 7.25-7.30 (*m*, 1H), 6.73 (*m*, 1H), 3.72 (*s*, 1H), 3.24 (*s*, 1H), 0.90 (*s*, 9H); ^{13}C NMR (75 MHz, $CDCl_3$) δ 145.4, 138.7, 138.3, 133.2, 130.7, 129.3, 127.3, 127.0, 119.3, 117.9, 111.4, 84.4, 79.7, 55.3, 29.6.

N-(*tert*-Butyl)-2-(2-ethynylphenyl)imidazo[1,2-*a*]pyrazin-3-amine (**4e**)

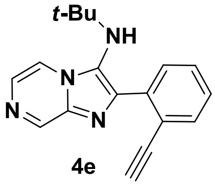

4e

Yield: 96%; ^{1}H NMR (400 MHz, $CDCl_3$) δ 9.00 (*d*, *J* = 1.2 Hz, 1H), 8.21-8.22 (*m*, 1H), 7.85-7.86 (*m*, 1H), 7.70 (*d*, *J* = 8.0 Hz, 1H), 7.61 (*d*, *J* = 8.0 Hz, 1H), 7.48-7.7.52 (*m*, 1H), 7.36-7.40 (*m*, 1H), 3.72 (*s*, 1H), 3.20 (*s*, 1H), 0.91 (*s*, 9H); ^{13}C NMR (100 MHz, $CDCl_3$) δ 143.5, 141.2, 137.6, 137.5, 133.4, 131.1, 130.9, 129.6, 128.9, 128.8, 128.1, 126.8, 120.3, 116.6, 83.9, 80.2, 56.4, 29.7.

N-(*tert*-Butyl)-2-(2-ethynyl-5-methoxyphenyl)imidazo[1,2-*a*]pyridin-3-amine (**4f**)

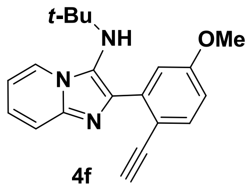

Yield: 89%; ^{1}H NMR (400 MHz, $CDCl_3$) δ 8.32 (*dd*, *J* = 1.2, 6.8 Hz, 1H), 7.48-7.54 (*m*, 2H), 7.33 (*d*, *J* = 2.4 Hz, 1H), 7.12-7.15 (*m*, 1H), 6.87-6.89 (*m*, 1H), 6.76 (*t*, *J* = 6.8 Hz, 1H), 3.87(*s*, 3H), 3.73 (*s*, 1H), 3.11 (*s*, 1H), 0.91 (*s*, 9H); ^{13}C NMR (100 MHz, $CDCl_3$) δ 160.2, 142.2, 140.4, 138.4, 134.7, 125.3, 124.2, 123.8, 117.3, 115.0, 114.7, 112.5, 111.2, 84.6, 78.3, 55.9, 55.5, 29.8.

N-(*tert*-Butyl)-2-(2-ethynyl-5-methoxyphenyl)-8-methylimidazo[1,2-*a*]pyridin-3-amine (**4g**)

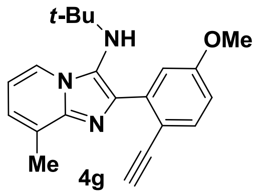

Yield: 61%; ^{1}H NMR (300 MHz, $CDCl_3$) δ 8.20 (*d*, *J* = 6.6 Hz, 1H), 7.49 (*d*, *J* = 8.4 Hz, 1H), 7.37 (*d*, *J* = 2.7 Hz, 1H), 6.95 (*d*, *J* = 2.7 Hz, 1H), 6.85(*dd*, *J* = 2.7, 8.9 Hz, 1H), 6.69 (*t*, *J* = 6.9 Hz, 1H), 3.89 (*s*, 3H), 3.72 (*s*, 1H), 3.11 (*s*, 1H), 2.63 (*s*, 3H), 0.92 (*s*, 9H); ^{13}C NMR (75 MHz, $CDCl_3$) δ 160.1, 142.4, 140.5, 137.8, 134.6, 126.9, 125.6, 123.0, 121.7, 115.1, 114.6, 112.5, 111.2, 84.5, 78.2, 55.8, 55.4, 29.8, 16.6.

N-(*tert*-Butyl)-6-(2-ethynyl-5-methoxyphenyl)imidazo[2,1-*b*]thiazol-5-amine (**4h**)

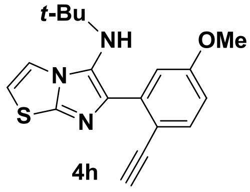

Yield: 85%; ^{1}H NMR (400 MHz, $CDCl_3$) δ 7.44 (*d*, *J* = 8.8 Hz, 1H), 7.42 (*d*, *J* = 4.4 Hz, 1H), 7.25 (*d*, *J* = 2.4 Hz, 1H), 6.84 (*dd*, *J* = 2.4, 8.4 Hz, 1H), 6.73 (*d*, *J* = 4.4 Hz, 1H), 3.86 (*s*, 3H), 3.80 (*s*, 1H), 3.17 (*s*, 1H), 0.92 (*s*, 9H); ^{13}C NMR (100 MHz, $CDCl_3$) δ 160.2, 145.4, 140.1, 138.8, 134.8, 127.4, 118.0, 114.7, 114.3, 111.7, 111.4, 84.7, 78.5, 55.5, 55.4, 29.7.

N-(*tert*-Butyl)-2-(2-ethynyl-5-methoxyphenyl)imidazo[1,2-*a*]pyrazin-3-amine (**4i**)

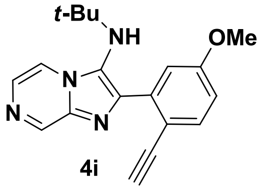

Yield: 88%; ^{1}H NMR (300 MHz, $CDCl_3$) δ 9.01 (*s*, 1H), 8.22 (*d*, *J* =4.5 Hz, 1H), 7.86 (*d*, *J* = 4.8 Hz, 1H), 7.52 (*d*, *J* = 8.4 Hz, 1H), 7.30 (*d*, *J* = 2.4 Hz, 1H), 6.93 (*dd*, *J* = 2.4, 8.7 Hz, 1H), 3.88 (*s*, 3H), 3.82 (*s*, 1H), 3.13 (*s*, 1H), 0.92 (*s*, 9H); ^{13}C NMR (75 MHz, $CDCl_3$) δ 160.3, 143.4, 141.1, 139.0, 137.4, 134.9, 128.6, 126.8, 116.7, 115.5, 114.9, 112.5, 84.0, 78.8, 56.4, 55.5, 29.7.

6-Bromo-*N*-(*tert*-butyl)-2-(2-ethynyl-4-methylphenyl)imidazo[1,2-*a*]pyridin-3-amine (**4j**)

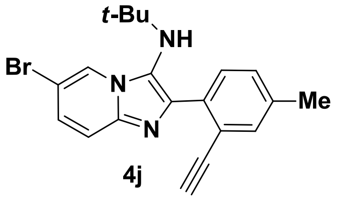

Yield: 64%; ^{1}H NMR (300 MHz, $CDCl_3$) δ 8.44 (*s*, 1H), 7.68 (*d*, *J* = 7.8 Hz, 1H), 7.43 (*d*, *J* = 9.3 Hz, 2H), 7.30 (*m*, 1H), 7.19 (*dd*, *J* = 1.8, 9.3 Hz, 1H), 3.68 (*s*, 1H), 3.16 (*s*, 1H), 2.38 (*s*, 3H), 0.91 (*s*, 9H); ^{13}C NMR (75 MHz, $CDCl_3$) δ 140.4, 139.3, 137.5, 135.2, 133.7, 130.8, 130.5, 127.3, 125.4, 123.8, 119.8, 118.0, 106.2, 84.4, 79.4, 55.9, 29.7, 21.0.

N-(*tert*-Butyl)-2-(2-ethynyl-4-methylphenyl)-6-methylimidazo[1,2-*a*]pyridin-3-amine (**4k**)

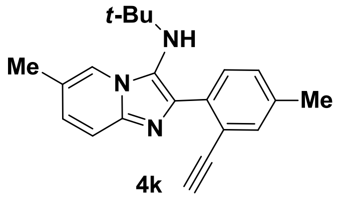

Yield: 75%; ^{1}H NMR (400 MHz, $CDCl_3$) δ 8.08 (*s*, 1H), 7.70 (*d*, *J* = 8.0 Hz, 1H), 7.39-7.44 (*m*, 2H), 7.28 (*d*, *J* = 8.8 Hz, 1H), 6.98 (*dd*, *J* = 1.6, 9.2 Hz, 1H), 3.65 (*s*, 1H), 3.14 (*s*, 1H), 2.37 (*s*, 3H), 2.33 (*s*, 3H), 0.91 (*s*, 9H); ^{13}C NMR (100 MHz, $CDCl_3$) δ 141.3, 138.3, 137.1, 136.0, 133.6, 130.9, 130.4, 127.2, 124.8, 121.3, 120.6, 119.7, 116.6, 84.7, 79.1, 55.8, 29.8, 21.0, 18.4.

N-(*tert*-Butyl)-2-(2-ethynyl-4-methylphenyl)-8-methylimidazo[1,2-*a*]pyridin-3-amine (**4l**)

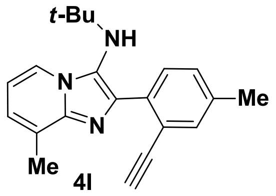

Yield: 49%; ^{1}H NMR (300 MHz, $CDCl_3$) δ 8.19 (*d*, *J* = 6.9 Hz, 1H), 7.74 (*d*, *J* = 8.1 Hz, 1H), 7.41 (*s*, 1H), 7.27-7.31 (*m*, 1H), 6.94 (*d*, *J* = 6.6 Hz, 1H), 6.69 (*t*, *J* = 6.6 Hz, 1H), 3.66 (*s*, 1H), 3.15 (*s*, 1H), 2.63 (*s*, 3H), 2.38 (*s*, 3H), 0.91 (*s*, 9H); ^{13}C NMR (75 MHz, $CDCl_3$) δ 142.4, 137.8, 137.1, 135.9, 133.5, 131.1, 130.5, 126.8, 125.4, 122.8, 121.6, 129.8, 111.1, 84.6, 79.1, 55.7, 29.8, 20.9, 16.7.

N-(*tert*-Butyl)-2-(5-chloro-2-ethynylphenyl)imidazo[1,2-*a*]pyridin-3-amine (**4m**)

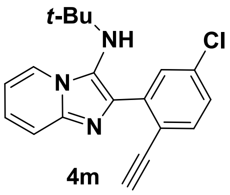

Yield: 71%; ^{1}H NMR (400 MHz, $CDCl_3$) δ 8.31 (*d*, *J* = 6.8 Hz, 1H), 7.86 (*d*, *J* = 2.0 Hz, 1H), 7.50-7.54 (*m*, 2H), 7.29-7.32 (*m*, 1H), 7.14-7.18 (*m*, 1H), 6.77-6.80 (*m*, 1H), 3.61 (*s*, 1H), 3.23 (*s*, 1H), 0.92 (*s*, 9H); ^{13}C NMR (100 MHz, $CDCl_3$) δ 142.4, 140.3, 137.2, 134.5, 131.0, 127.7, 125.6, 124.5, 123.8, 118.5, 117.4, 111.4, 83.5, 80.5, 56.0, 29.8.

N-(*tert*-Butyl)-2-(5-chloro-2-ethynylphenyl)-8-methylimidazo[1,2-*a*]pyridin-3-amine (**4n**)

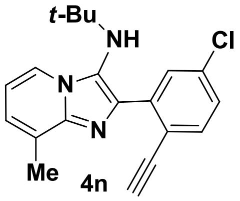

Yield: 47%; ^{1}H NMR (300 MHz, $CDCl_3$) δ 8.19 (*d*, *J* = 6.9 Hz, 1H), 7.88 (*d*, *J* = 2.1 Hz, 1H), 7.51 (*d*, *J* = 8.1 Hz, 1H), 7.30 (*dd*, *J* = 2.1, 8.1 Hz, 1H), 6.97 (*d*, *J* = 6.9 Hz, 1H), 6.71 (*t*, *J* = 6.9 Hz, 1H), 3.59 (*s*, 1H), 3.22 (*s*,1H), 2.62 (*s*, 3H), 0.92 (*s*, 9H); ^{13}C NMR (75 MHz, $CDCl_3$) δ 142.6, 140.5, 136.6, 135.4, 134.3, 131.2, 127.6, 127.0, 125.8, 123.2, 121.7, 118.5, 114.4, 83.4, 80.4, 55.8, 29.8, 16.6.

N-(*tert*-Butyl)-6-(5-chloro-2-ethynylphenyl)imidazo[2,1-*b*]thiazol-5-amine (**4o**)

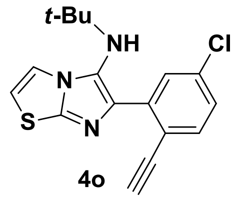

4o

Yield: 54%; ^{1}H NMR (400 MHz, $CDCl_3$) δ 7.77 (*d*, *J* = 2.0 Hz, 1H), 7.48 (*d*, *J* = 8.4 Hz, 1H), 7.43 (*d*, *J* = 4.4 Hz, 1H), 7.24-7.27 (*m*, 1H), 6.76 (*d*, *J* = 4.4 Hz, 1H), 3.69 (*s*, 1H), 3.28 (*s*, 1H), 0.92 (*s*, 9H); ^{13}C NMR (100 MHz, $CDCl_3$) δ 145.8, 139.9, 137.5, 135.4, 134.5, 130.7, 127.7, 127.4, 118.0, 117.7, 111.9, 83.6, 80.7, 55.5, 29.7.

N-(*tert*-Butyl)-2-(5-chloro-2-ethynylphenyl)imidazo[1,2-*a*]pyrazin-3-amine (**4p**)

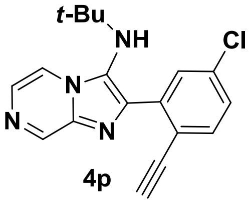

4p

Yield: 95%; ^{1}H NMR (400 MHz, $CDCl_3$) δ 8.99 (*d*, *J* = 1.6 Hz, 1H), 8.20-8.21 (*m*, 1H), 7.87 (*dd*, *J* = 2.4, 4.8 Hz, 1H), 7.82 (*t*, *J* = 2.0 Hz, 1H), 7.53 (*dd*, *J* = 2.0, 8.4 Hz, 1H), 7.33-7.36 (*m*, 1H), 3.67 (*s*, 1H), 3.24 (*s*, 1H), 0.92 (*s*, 9H); ^{13}C NMR (100 MHz, $CDCl_3$) δ 143.7, 139.9, 139.2, 137.6, 135.7, 134.6, 131.0, 128.9, 128.5, 127.0, 118.7, 116.7, 83.0, 81.1, 56.5, 29.8.

N-(*tert*-Butyl)-2-(2-ethynyl-4-fluorophenyl)-6-methylimidazo[1,2-*a*]pyridin-3-amine (**4q**)

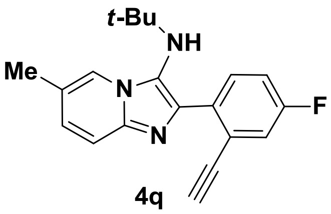

4q

Yield: 74%; ^{1}H NMR (300 MHz, $CDCl_3$) δ 8.09 (*s*, 1H), 7.76-7.81 (*m*, 1H), 7.43 (*d*, *J* = 9.0 Hz, 1H), 7.28 (*dd*, *J* = 2.4, 9.0 Hz, 1H), 7.16-7.23 (*m*, 1H), 7.01 (*dd*, *J* = 1.2, 8.4 Hz, 1H), 3.52 (*s*, 1H), 3.20 (*s*, 1H), 2.35 (*s*, 3H), 0.91 (*s*, 9H); ^{13}C NMR (75 MHz, $CDCl_3$) δ 163.2, 159.9, 141.3, 137.4, 135.1, 132.9, 132.8, 127.4, 124.8, 121.4, 121.3, 120.8, 119.6, 119.2, 117.2, 116.9, 116.6, 83.1, 80.3, 55.8, 29.7, 18.4.

N-(*tert*-Butyl)-2-(2-ethynyl-4-fluorophenyl)-8-methylimidazo[1,2-*a*]pyridin-3-amine (**4r**)

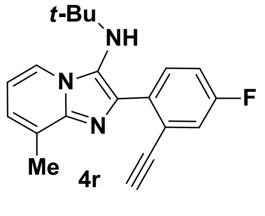

4r

Yield: 43%; ^{1}H NMR (300 MHz, $CDCl_3$) δ 8.19 (*d*, *J* = 6.6 Hz, 1H), 7.85 (*dd*, *J* = 6.0, 8.4 Hz, 1H), 7.17-7.30 (*m*, 2H), 6.95 (*d*, *J* = 6.3 Hz, 1H), 6.70 (*t*, *J* = 6.6 Hz, 1H), 3.53 (*s*, 1H), 3.20 (*s*, 1H), 2.62 (*s*, 3H), 0.91 (*s*, 9H); ^{13}C NMR (75 MHz, $CDCl_3$) δ 163.2, 159.9, 142.5, 137.0, 135.2, 133.2, 133.1, 126.9, 125.5, 123.0, 121.6, 121.5, 119.5, 119.2, 117.3, 117.0, 111.3, 83.0, , 80.3, 55.7, 29.8, 16.6.

N-(*tert*-Butyl)-6-(2-ethynyl-5-fluorophenyl)imidazo[2,1-*b*]thiazol-5-amine (**4s**)

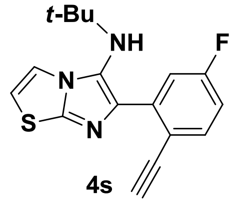

4s

Yield: 57%; ^{1}H NMR (400 MHz, $CDCl_3$) δ 7.42-7.55 (*m*, 3H), 6.97-7.01 (*m*, 1H), 6.75 (*d*, *J* = 4.8 Hz, 1H), 3.72-3.75 (*m*, 1H), 3.22 (*s*, 1H), 0.92 (*s*, 9H); ^{13}C NMR (100 MHz, $CDCl_3$) δ 164.1, 161.6, 145.8, 140.9, 140.8, 137.7, 135.4, 135.3, 118.0, 117.6, 117.3, 115.4, 114.8, 114.6, 111.8, 83.6, 79.6, 55.5, 29.7.

5-Methylpyrido[2',1':2,3]imidazo[4,5-*c*]isoquinoline (**6a**)

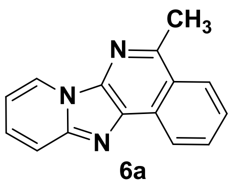

6a

Yield = 83%; ^{1}H NMR (300 MHz, $CDCl_3$) δ 8.93 (*d*, *J* = 6.9 Hz, 1H), 8.81 (*d*, *J* = 8.1 Hz, 1H), 8.33 (*d*, *J* = 8.4 Hz, 2H), 7.82-7.94 (*m*, 2H), 7.70-7.75 (*m*, 1H), 7.47-7.53 (*m*, 1H), 7.05 (*t*, *J* = 6.6 Hz, 1H), 3.14 (*s*, 3H); ^{13}C NMR (75 MHz, $CDCl_3$) δ 152.6, 130.2, 129.7, 128.3, 126.8, 126.3, 123.7, 122.6, 117.7, 111.3, 22.8; HRMS (*m/z*) calc. for C15H12N3 (+) 234.1031, found 234.1025.

5,9-Dimethylpyrido[2',1':2,3]imidazo[4,5-*c*]isoquinoline (**6b**)

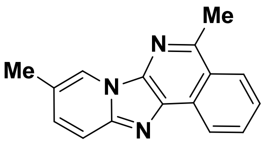

6b

Yield = 72%; ^{1}H NMR (300 MHz, $CDCl_3$) δ 8.79 (*d*, *J* = 8.1 Hz, 1H), 8.69 (*s*, 1H), 8.30 (*d*, *J* = 8.4 Hz, 1H), 7.91 (*t*, *J* = 7.2 Hz, 1H), 7.67-7.86 (*m*, 2H), 7.28-7.36 (*m*, 1H), 3.11 (*s*, 3H), 2.47 (*s*, 3H); ^{13}C NMR (75 MHz, $CDCl_3$) δ 152.3, 145.0, 133.1, 131.7, 131.1, 130.2, 129.6, 128.9, 126.8, 122.6, 121.3, 121.2, 116.9, 22.8, 18.1; HRMS (*m/z*) calc. for C16H14N3 (+) 248.1188, found 248.1183.

9-Chloro-5-methylpyrido[2',1':2,3]imidazo[4,5-*c*]isoquinoline (**6c**)

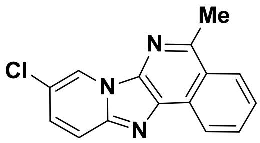

6c

Yield = 75%; ^{1}H NMR (300 MHz, $CDCl_3$) δ 8.92 (*d*, *J* = 1.2 Hz, 1H), 8.75 (*d*, *J* = 8.1 Hz, 1H), 8.30 (*d*, *J* = 8.4 Hz, 1H), 7.87-7.92 (*m*, 1H), 7.69-7.76 (*m*, 2H), 7.43 (*dd*, *J* = 1.8, 9.6 Hz, 1H), 3.12 (*s*, 3H); ^{13}C NMR (100 MHz, $CDCl_3$) δ 153.6, 144.0, 131.8, 130.5, 129.5, 128.9, 127.0, 126.3, 122.6, 121.7, 119.7, 118.1, 109.5, 22.8; HRMS (*m/z*) calc. for C15H11ClN3 (+) 268.0642, found 268.0634.

5-Methylthiazolo[2',3':2,3]imidazo[4,5-*c*]isoquinoline (**6d**)

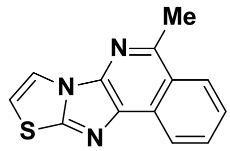

6d

Yield = 61%; ^{1}H NMR (300 MHz, $CDCl_3$) δ 8.63 (*d*, *J* = 8.1 Hz, 1H), 8.26 (*d*, *J* = 8.4 Hz, 1H), 8.06 (*d*, *J* = 3.9 Hz, 1H), 7.88 (*t*, *J* = 7.2 Hz, 1H), 7.66 (*t*, *J* = 7.5 Hz, 1H), 6.97 (*d*, *J* = 3.6 Hz, 1H), 3.08 (*s*, 3H); ^{13}C NMR (75 MHz, $CDCl_3$) δ 151.8, 130.2, 129.7, 126.5, 125.5, 121.9, 117.1, 111.7, 22.5; HRMS (*m/z*) calc. for C13H10N3S (+) 240.0595, found 240.0593.

5-Methylpyrazino[2',1':2,3]imidazo[4,5-*c*]isoquinoline (**6e**)

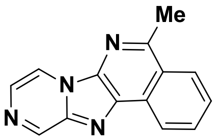

6e

Yield = 63%; ^{1}H NMR (300 MHz, $CDCl_3$) δ 9.37 (*d*, *J* = 1.5 Hz, 1H), 8.74-8.82 (*m*, 2H), 8.34 (*d*, *J* = 8.4 Hz, 1H), 8.12 (*d*, *J* = 4.5 Hz, 1H), 7.90-8.00 (*m*, 1H), 7.78 (*t*, *J* = 6.9 Hz, 1H), 3.13 (*s*, 3H); ^{13}C NMR (75 MHz, $CDCl_3$) δ 156.1, 144.8, 141.8, 139.8, 131.0, 130.0, 128.4, 127.4, 127.1, 122.8, 116.3, 23.0; HRMS (*m/z*) calc. for C14H11N4 (+) 235.0984, found 235.0971.

2-Methoxy-5-methylpyrido[2',1':2,3]imidazo[4,5-*c*]isoquinoline (**6f**)

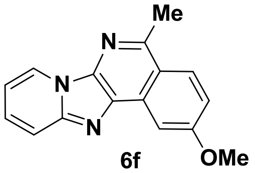

6f

Yield = 78%; ^{1}H NMR (300 MHz, $CDCl_3$) δ 8.90 (*d*, *J* = 6.6 Hz, 1H), 8.21 (*d*, *J* = 9.3 Hz, 1H), 8.08 (*d*, *J* = 2.4 Hz, 1H), 7.85 (*d*, *J* = 9.0 Hz, 1H), 7.50-7.70 (*m*, 2H), 6.99-7.03 (*m*, 1H), 4.09 (*s*, 3H), 3.08 (*s*, 3H); ^{13}C NMR (75 MHz, $CDCl_3$) δ 161.1, 130.8, 128.8, 128.7, 123.8, 121.9, 118.5, 117.6, 111.3, 101.1, 55.8, 22.7; HRMS (*m/z*) calc. for C16H14N3O (+) 264.1137, found 264.1128.

2-Methoxy-5,11-dimethylpyrido[2',1':2,3]imidazo[4,5-*c*]isoquinoline (**6g**)

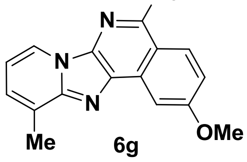

6g

Yield = 80%; ^{1}H NMR (300 MHz, $CDCl_3$) δ 8.77 (*d*, *J* = 6.9 Hz, 1H), 8.13-8.20 (*m*, 2H), 7.25-7.29 (*m*, 2H), 6.93 (*t*, *J* = 6.9 Hz, 1H), 4.12 (*s*, 3H), 3.07 (*s*, 3H), 2.80 (*s*, 3H); ^{13}C NMR (75 MHz, $CDCl_3$) δ 161.0, 152.0, 131.9, 128.64, 127.3, 126.7, 121.8, 118.2, 111.2, 101.4, 55.8, 22.7, 17.1; HRMS (*m/z*) calc. for C17H16N3O (+) 278.1293, found 278.1288.

2-Methoxy-5-methylthiazolo[2',3':2,3]imidazo[4,5-*c*]isoquinoline (**6h**)

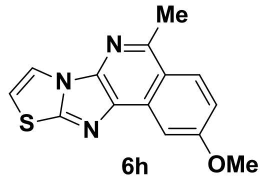

6h

Yield = 56%; ^{1}H NMR (300 MHz, $CDCl_3$) *δ* 8.12 (*d*, *J* = 9.3 Hz, 1H), 8.04 (*d*, *J* = 4.5 Hz, 1H), 7.85 (*d*, *J* = 2.1 Hz, 1H), 7.22 (*dd*, *J* = 2.4, 9.0 Hz, 1H), 6.93 (*d*, *J* = 4.5 Hz, 1H), 4.04 (*s*, 3H), 3.00 (*s*, 3H); ^{13}C NMR (75 MHz, $CDCl_3$) *δ* 161.0, 153.0, 151.4, 136.6, 133.7, 131.6, 128.4, 120.7, 118.0, 117.2, 111.6, 100.0, 55.7, 22.4; HRMS (*m/z*) calc. for C14H12N3OS (+) 270.0701, found 270.0694.

2-Methoxy-5-methylpyrazino[2',1':2,3]imidazo[4,5-*c*]isoquinoline (**6i**)

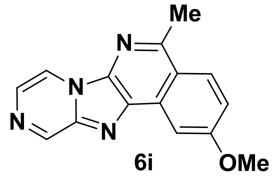

6i

Yield = 58%; ^{1}H NMR (300 MHz, $CDCl_3$) *δ* 9.35 (*d*, *J* = 1.5 Hz, 1H), 8.74 (*dd*, *J* = 1.5, 4.5 Hz, 1H), 8.23 (*d*, *J* = 9.0 Hz, 1H), 8.07-8.10 (*m*, 2H), 7.36 (*dd*, *J* = 2.7, 9.0 Hz, 1H), 4.10 (*s*, 3H), 3.08 (*s*, 3H); ^{13}C NMR (75 MHz, $CDCl_3$) *δ* 161.7, 155.6, 144.7, 139.5, 132.21, 132.0, 129.0, 128.2, 122.3, 119.1, 116.4, 101.6, 55.9, 22.9; HRMS (*m/z*) calc. for C15H13N4O (+) 265.1089, found 265.1083.

9-Bromo-3,5-dimethylpyrido[2',1':2,3]imidazo[4,5-*c*]isoquinoline (**6j**)

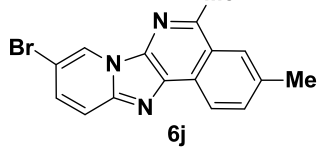

6j

Yield = 78%; ^{1}H NMR (400 MHz, $CDCl_3$) *δ* 8.97 (*s*, 1H), 8.62 (*d*, *J* = 8.4 Hz, 1H), 8.03 (*s*, 1H), 7.68-7.73 (*m*, 2H), 7.47-7.50 (*m*, 1H), 3.07 (*s*, 3H), 2.64 (*s*, 3H); ^{13}C NMR (75 MHz, $CDCl_3$) *δ* 153.1, 143.9, 136.5, 133.8, 132.4, 131.6, 131.2, 127.5, 127.1, 126.1, 123.8, 122.4, 118.3, 105.7, 22.8, 22.1; HRMS (*m/z*) calc. for $C_{16}H_{13}BrN_3$ (+) 326.0293, found 326.0300.

3,5,9-Trimethylpyrido[2',1':2,3]imidazo[4,5-*c*]isoquinoline (**6k**)

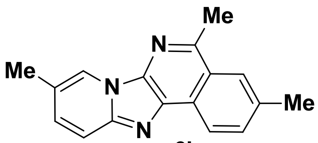

6k

Yield = 87%; ^{1}H NMR (400 MHz, $CDCl_3$) δ 8.64 (*s*, 1H), 8.02 (*s*, 1H), 7.71 (*t*, J = 8.8 Hz, 2H), 7.31 (*d*, J = 9.2 Hz, 1H), 3.07 (*s*, 3H), 2.63 (*s*, 3H), 2.45 (*s*, 3H); ^{13}C NMR (100 MHz, $CDCl_3$) δ 151.7, 145.0, 135.9, 134.1, 132.1, 131.4, 131.3, 127.6, 126.9, 126.0, 122.4, 121.2, 120.9, 116.9, 22.8, 22.0, 18.1; HRMS (*m/z*) calc. for $C_{17}H_{16}N_3$ (+) 262.1344, found 262.1341.

3,5,11-Trimethylpyrido[2',1':2,3]imidazo[4,5-*c*]isoquinoline (**6l**)

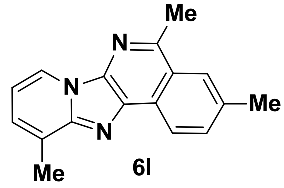

6l

Yield = 79%; ^{1}H NMR (300 MHz, $CDCl_3$) δ 8.73 (*m*, 2H), 8.03 (*s*, 1H), 7.71 (*dd*, J = 1.2, 8.4 Hz, 1H), 7.25 (*d*, J = 6.6 Hz, 1H), 6.90 (*t*, J = 6.6 Hz, 1H), 3.09 (*s*, 3H), 2.79 (*s*, 3H), 2.64 (*s*, 3H); ^{13}C NMR (75 MHz, $CDCl_3$) δ 151.8, 146.3, 135.9, 134.7, 131.9, 131.0, 129.1, 128.6, 127.7, 127.3, 126.7, 122.6, 121.4, 111.2, 22.8, 22.0, 17.1; HRMS (*m/z*) calc. for $C_{17}H_{16}N_3$ (+) 262.1344, found 262.1335.

2-Chloro-5-methylpyrido[2',1':2,3]imidazo[4,5-*c*]isoquinoline (**6m**)

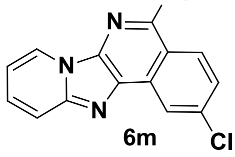

6m

Yield = 48%; ^{1}H NMR (300 MHz, $CDCl_3$) δ 8.89 (*d*, J = 6.6 Hz, 1H), 8.76 (*s*, 1H), 8.22 (*d*, J = 9.0 Hz, 1H), 7.86 (*d*, J = 6.9 Hz, 1H), 7.63 (*d*, J = 8.7 Hz, 1H), 7.51-7.56 (*m*, 1H), 7.05 (*t*, J = 6.6 Hz, 1H), 3.10 (*s*, 3H); ^{13}C NMR (75 MHz, $CDCl_3$) δ 136.7, 129.1, 128.7, 128.6, 128.4, 127.0, 125.0, 123.8, 122.0, 117.8, 111.6, 22.7; HRMS (*m/z*) calc. for $C_{15}H_{11}ClN_3$ (+) 268.0642, found 268.0635.

2-Chloro-5,11-dimethylpyrido[2',1':2,3]imidazo[4,5-*c*]isoquinoline (**6n**)

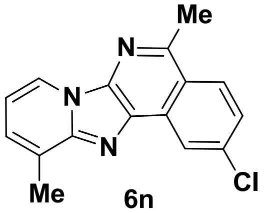

6n

Yield = 55%; ^{1}H NMR (300 MHz, $CDCl_3$) δ 8.80-8.72 (*m*, 2H), 8.17 (*d*, J = 7.5 Hz, 1H), 7.28-7.60 (*m*, 2H), 6.92 (*s*, 1H), 3.07 (*s*, 3H), 2.78 (*s*, 3H); ^{13}C NMR (75 MHz, $CDCl_3$) δ 152.1, 146.8, 136.4, 135.5, 130.6, 130.4, 128.3, 127.6, 127.1, 126.8, 124.8, 122.0, 121.4, 111.5, 22.7, 17.1; HRMS (*m/z*) calc. for $C_{16}H_{13}ClN_3$ (+) 282.0798, found 282.0791.

2-Chloro-5-methylthiazolo[2',3':2,3]imidazo[4,5-*c*]isoquinoline (**6o**)

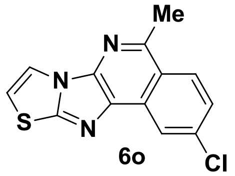

6o

Yield = 62%; ^{1}H NMR (300 MHz, $CDCl_3$) δ 8.56 (*d*, J = 1.8 Hz, 1H), 8.15 (*d*, J = 9.0 Hz, 1H), 8.03 (*d*, J = 4.5 Hz, 1H), 7.55 (*dd*, J = 2.4, 8.7 Hz, 1H), 6.98 (*d*, J = 4.5 Hz, 1H), 3.03 (*s*, 3H); ^{13}C NMR (75 MHz, $CDCl_3$) δ 153.8, 151.6, 136.7, 136.6, 133.1, 130.3, 128.2, 123.6, 121.1, 117.0, 112.1, 22.4; HRMS (*m/z*) calc. for C13H9ClN3S (+) 274.0206, found 274.0198.

2-Chloro-5-methylpyrazino[2',1':2,3]imidazo[4,5-*c*]isoquinoline (**6p**)

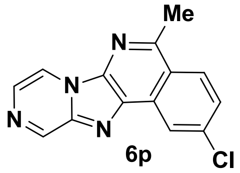

6p

Yield = 63%; ^{1}H NMR (300 MHz, $CDCl_3$) δ 9.39 (*d*, J = 1.2 Hz, 1H), 8.76-8.82 (*m*, 2H), 8.30 (*d*, J = 9.0 Hz, 1H), 8.15 (*d*, J = 4.5 Hz, 1H), 7.75 (*dd*, J = 2.4, 9.0 Hz, 1H), 3.13 (*s*, 3H); ^{13}C NMR (75 MHz, $CDCl_3$) δ 145.0, 128.8, 128.5, 128.1, 122.3, 116.4, 29.2; HRMS (*m/z*) calc. for C14H10ClN4 (+) 269.0594, found 269.0592.

3-Fluoro-5,9-dimethylpyrido[2',1':2,3]imidazo[4,5-*c*]isoquinoline (**6q**)

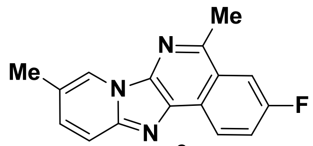

6q

Yield = 58%; ^{1}H NMR (300 MHz, $CDCl_3$) *δ* 8.71 (*d*, *J* = 8.1 Hz, 1H), 8.61 (*s*, 1H), 7.81 (*d*, *J* = 10.2 Hz, 1H), 7.70 (*d*, *J* = 9.0 Hz, 1H), 7.59 (*t*, *J* = 7.8 Hz, 1H), 7.33 (*d*, *J* = 9.0 Hz, 1H), 3.02 (*s*, 3H), 2.45 (*s*, 3H); ^{13}C NMR (75 MHz, $CDCl_3$) *δ* 162.0, 158.8, 151.0, 145.2, 134.2, 131.8, 128.8, 127.6, 126.3, 125.0, 121.2, 119.9, 116.9, 110.9, 22.7, 18.1; HRMS (*m/z*) calc. for $C_{16}H_{13}FN_3$ (+) 266.1094 found 266.1091.

3-Fluoro-5,11-dimethylpyrido[2',1':2,3]imidazo[4,5-*c*]isoquinoline (**6r**)

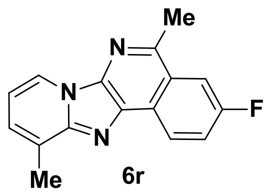

6r

Yield = 67%; ^{1}H NMR (300 MHz, $CDCl_3$) *δ* 8.82 (*dd*, *J* = 5.7, 9.0 Hz, 1H), 8.75 (*d*, *J* = 6.6 Hz, 1H), 7.86 (*dd*, *J* = 2.4, 10.5 Hz, 1H), 7.59-7.66 (*m*, 1H), 7.14 (*d*, *J* = 6.9 Hz, 1H), 6.93 (*t*, *J* = 6.9 Hz, 1H), 3.06 (*s*, 3H), 2.78 (*s*, 3H); ^{13}C NMR (75 MHz, $CDCl_3$) *δ* 151.2, 146.5, 129.1, 128.6, 128.1, 127.4, 126.4, 125.4, 121.5, 119.8, 119.5, 111.5, 110.9, 22.7, 17.1; HRMS (*m/z*) calc. for $C_{16}H_{13}FN_3$ (+) 266.1094, found 266.1089.

2-Fluoro-5-methylthiazolo[2',3':2,3]imidazo[4,5-*c*]isoquinoline (**6s**)

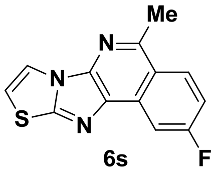

6s

Yield = 59%; ^{1}H NMR (300 MHz, $CDCl_3$) *δ* 8.60 (*dd*, *J* = 5.4, 8.7 Hz, 1H),, 8.05 (*d*, *J* = 4.5 Hz, 1H), 7.81-7.86 (*m*, 1H), 7.59-7.65 (*m*, 1H), 6.98 (*d*, *J* = 4.5 Hz, 1H), 3.02 (*s*, 3H); ^{13}C NMR (75 MHz, $CDCl_3$) *δ* 161.6, 158.3, 153.8, 150.6, 130.8, 129.8, 128.9, 126.5, 124.5, 120.4, 117.1, 111.9, 110.6, 22.5; HRMS (*m/z*) calc. for C13H9FN3S (+) 258.0501, found 258.0498.

^{1}H NMR and ^{13}C NMR spectra

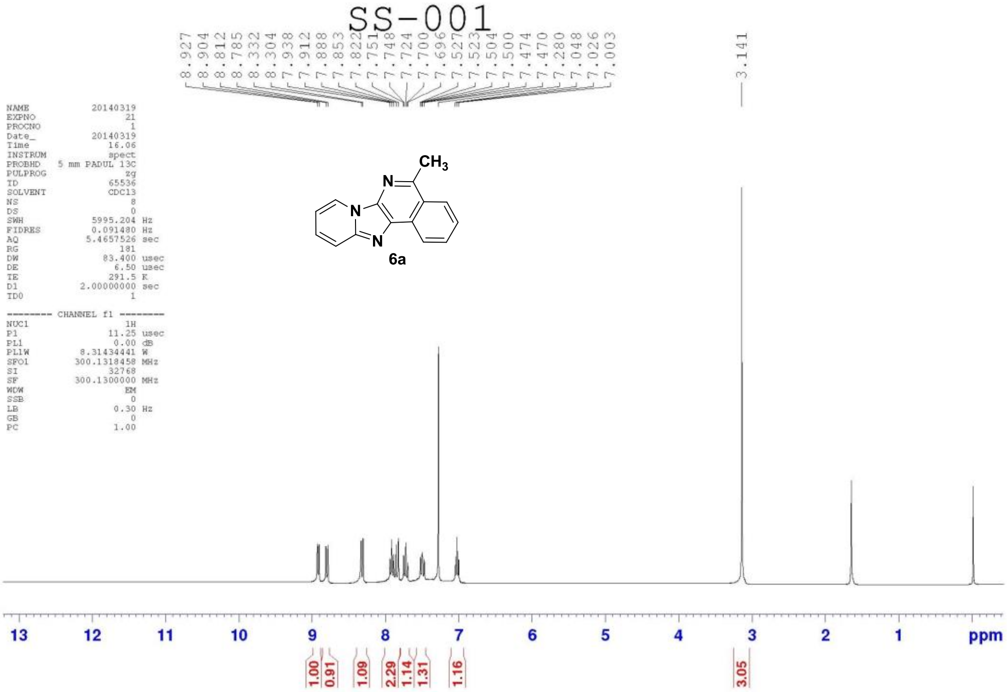

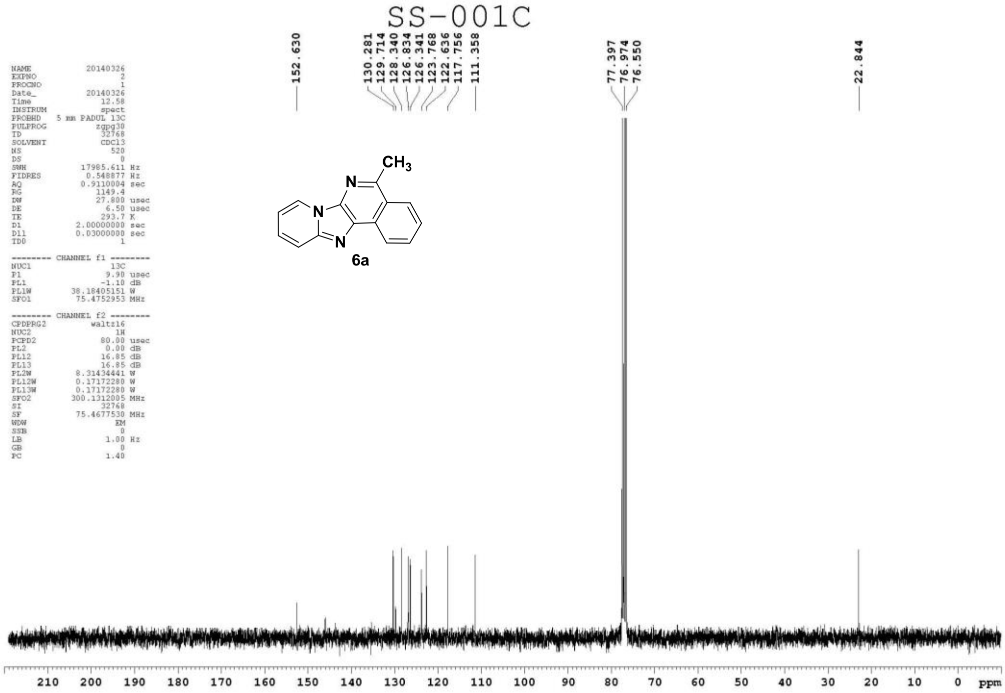

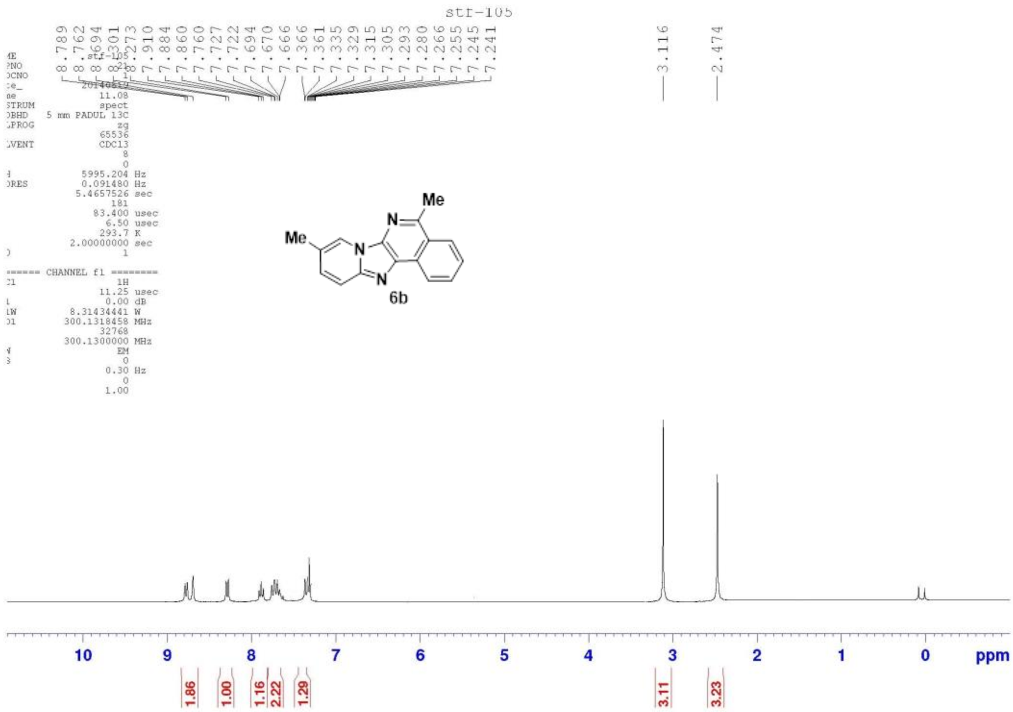
stf-105
8.789 8.762 8.694 8.301 8.273 7.910 7.884 7.860 7.760 7.727 7.722 7.694 7.670 7.666 7.366 7.361 7.335 7.329 7.315 7.305 7.293 7.280 7.266 7.255 7.245 7.241
3.116
2.474
Me
N
Me
N
N
6b
1.86 1.00 1.16 2.22 1.29 3.11 3.23
10 9 8 7 6 5 4 3 2 1 0 ppm

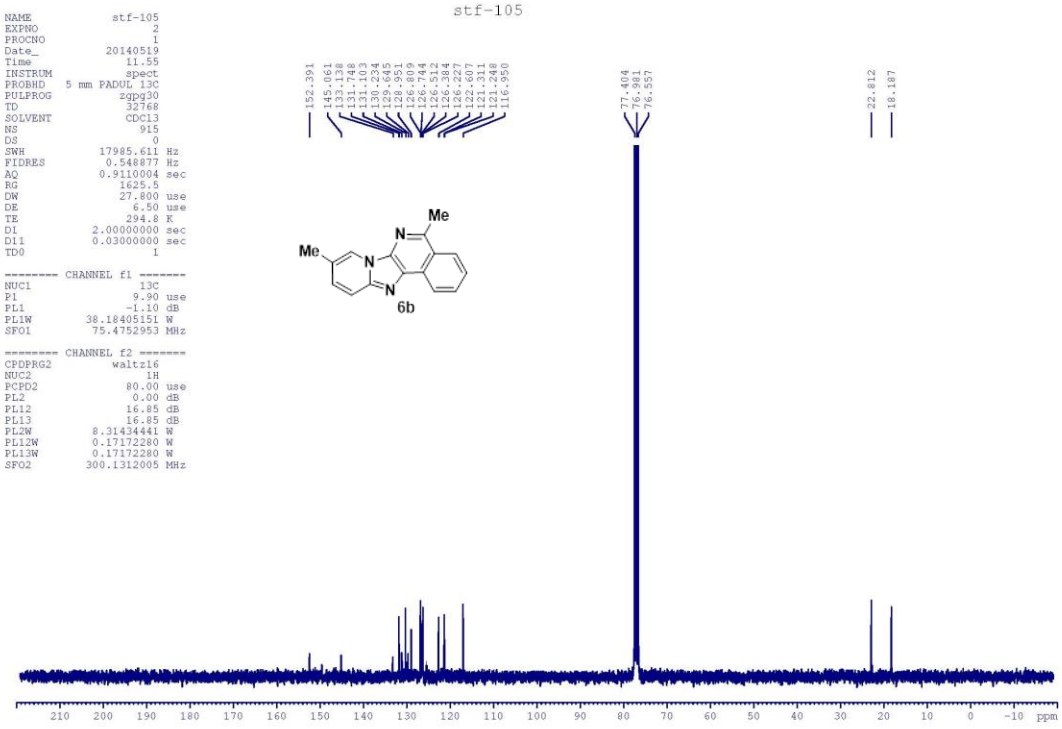
stf-105
NAME stf-105
EXPNO 2
PROCNO 1
Date_ 20140519
Time 11.55
INSTRUM spect
PROBHD 5 mm PADUL 13C
PULPROG zgpg30
TD 32768
SOLVENT CDCl3
NS 915
DS 0
SWH 17985.611 Hz
FIDRES 0.548877 Hz
AQ 0.9110004 sec
RG 1625.5
DW 27.800 use
DE 6.50 use
TE 294.8 K
D1 2.00000000 sec
D11 0.03000000 sec
TD0 1
======== CHANNEL f1 ========
NUC1 13C
P1 9.90 use
PL1 -1.10 dB
PL1W 38.18405151 W
SFO1 75.4752953 MHz
======== CHANNEL f2 ========
CPDPRG2 waltz16
NUC2 1H
PCPD2 80.00 use
PL2 0.00 dB
PL12 16.85 dB
PL13 16.85 dB
PL2W 8.31434441 W
PL12W 0.17172280 W
PL13W 0.17172280 W
SFO2 300.1312005 MHz
152.391 145.061 133.138 131.748 131.103 130.234 129.645 128.951 126.809 126.744 126.512 126.384 126.227 122.607 121.311 121.248 116.950
77.404 76.981 76.557
22.812 18.187
Me
N
Me
N
N
6b
210 200 190 180 170 160 150 140 130 120 110 100 90 80 70 60 50 40 30 20 10 0 -10 ppm

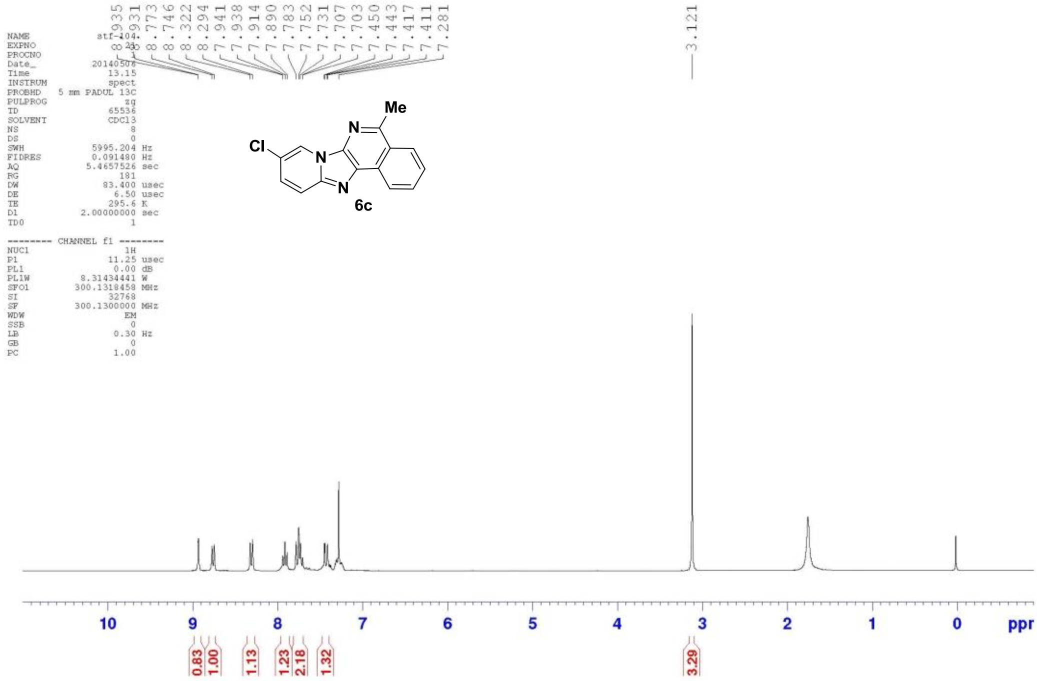

NAME stf-104
EXPNO 2
PROCNO 1
Date_ 20140506
Time 13.15
INSTRUM spect
PROBHD 5 mm PADUL 13C
PULPROG zg
TD 65536
SOLVENT CDCl3
NS 8
DS 0
SWH 5995.204 Hz
FIDRES 0.091480 Hz
AQ 5.4657526 sec
RG 181
DW 83.400 usec
DE 6.50 usec
TE 295.6 K
D1 2.00000000 sec
TD0 1
======== CHANNEL f1 ========
NUC1 1H
P1 11.25 usec
PL1 0.00 dB
PL1W 8.31434441 W
SFO1 300.1318458 MHz
SI 32768
SF 300.1300000 MHz
WDW EM
SSB 0
LB 0.30 Hz
GB 0
PC 1.00
8.935 8.931 8.773 8.746 8.322 8.294 7.941 7.938 7.914 7.890 7.783 7.752 7.731 7.707 7.703 7.450 7.443 7.417 7.411 7.281
3.121
Me
N
Cl
N
N
6c
10 9 8 7 6 5 4 3 2 1 0 ppm
0.83 1.00 1.13 1.23 2.18 1.32 3.29

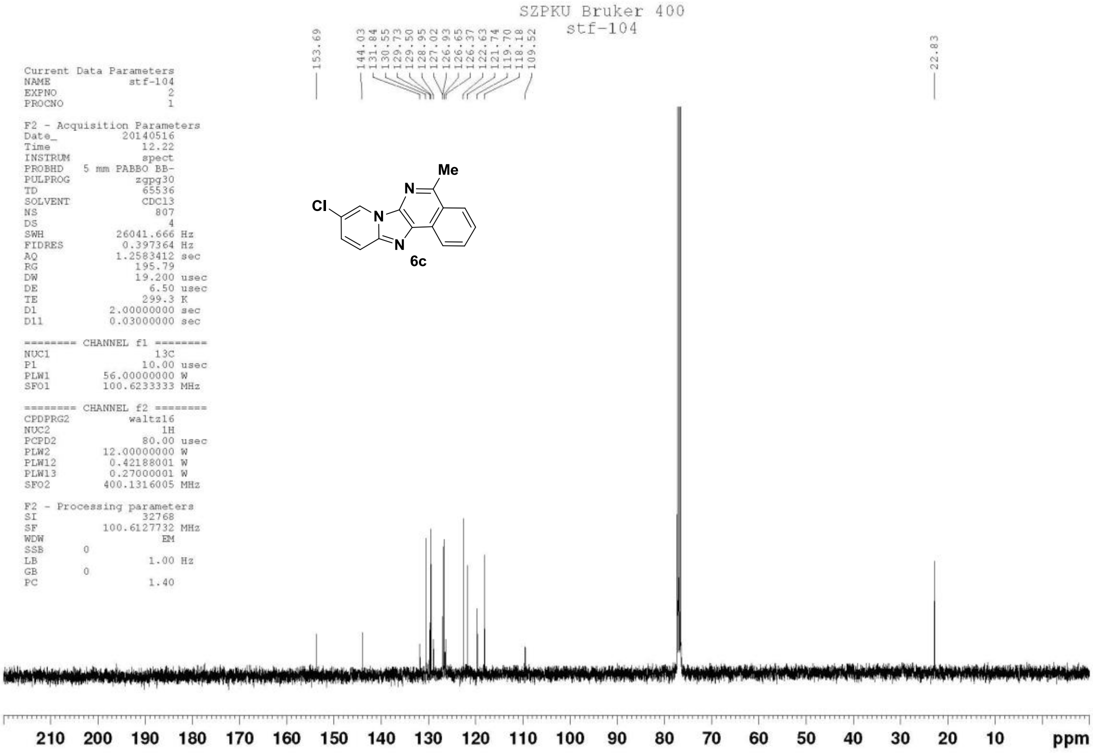

SZPKU Bruker 400
stf-104
Current Data Parameters
NAME stf-104
EXPNO 2
PROCNO 1
F2 - Acquisition Parameters
Date_ 20140516
Time 12.22
INSTRUM spect
PROBHD 5 mm PABBO BB-
PULPROG zgpg30
TD 65536
SOLVENT CDCl3
NS 807
DS 4
SWH 26041.666 Hz
FIDRES 0.397364 Hz
AQ 1.2583412 sec
RG 195.79
DW 19.200 usec
DE 6.50 usec
TE 299.3 K
D1 2.00000000 sec
D11 0.03000000 sec
======== CHANNEL f1 ========
NUC1 13C
P1 10.00 usec
PLW1 56.00000000 W
SFO1 100.6233333 MHz
======== CHANNEL f2 ========
CPDPRG2 waltz16
NUC2 1H
PCPD2 80.00 usec
PLW2 12.00000000 W
PLW12 0.42188001 W
PLW13 0.27000001 W
SFO2 400.1316005 MHz
F2 - Processing parameters
SI 32768
SF 100.6127732 MHz
WDW EM
SSB 0
LB 1.00 Hz
GB 0
PC 1.40
153.69
144.03 131.84 130.55 129.73 129.50 128.95 127.02 126.93 126.65 126.37 122.63 121.74 119.70 118.18 109.52
22.83
Me
N
Cl
N
N
6c
210 200 190 180 170 160 150 140 130 120 110 100 90 80 70 60 50 40 30 20 10 ppm

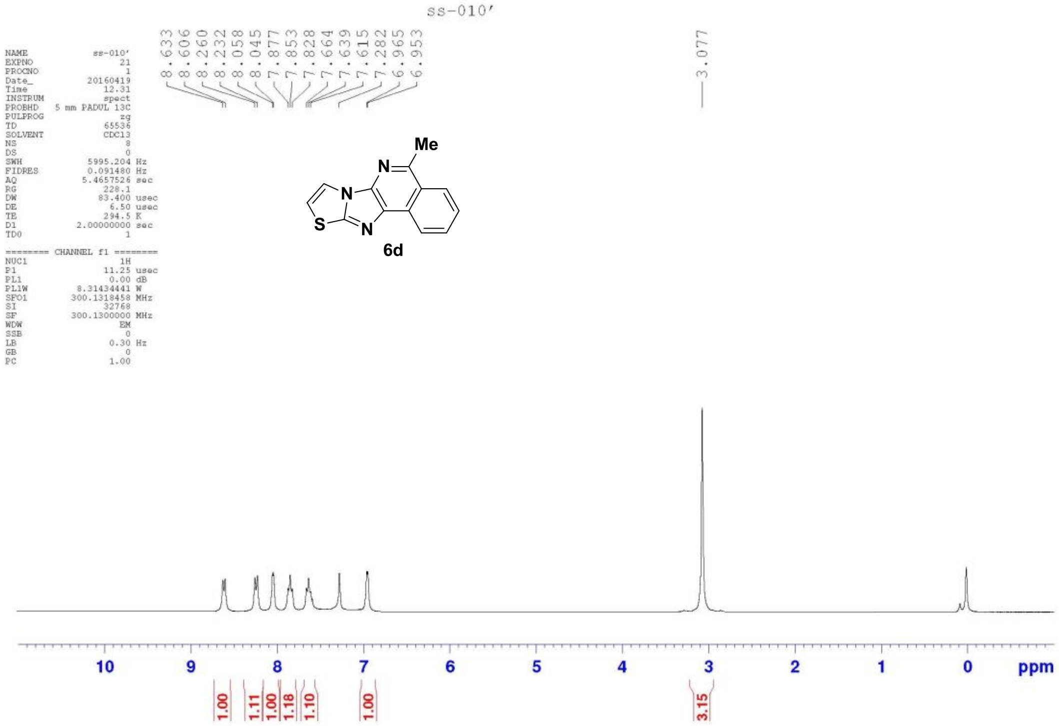
ss-010'
8.633
8.606
8.260
8.232
8.058
8.045
7.877
7.853
7.828
7.664
7.639
7.615
7.282
6.965
6.953
3.077
NAME ss-010'
EXPNO 21
PROCNO 1
Date_ 20160419
Time 12.31
INSTRUM spect
PROBHD 5 mm PADUL 13C
PULPROG zg
TD 65536
SOLVENT CDCl3
NS 8
DS 0
SWH 5995.204 Hz
FIDRES 0.091480 Hz
AQ 5.4657526 sec
RG 228.1
DW 83.400 usec
DE 6.50 usec
TE 294.5 K
D1 2.00000000 sec
TD0 1
======== CHANNEL f1 ========
NUC1 1H
P1 11.25 usec
PL1 0.00 dB
PL1W 8.31434441 W
SFO1 300.1318458 MHz
SI 32768
SF 300.1300000 MHz
WDW EM
SSB 0
LB 0.30 Hz
GB 0
PC 1.00
Me
N
N
S
N
6d
10
9
8
7
6
5
4
3
2
1
0
ppm
1.00
1.11
1.00
1.18
1.10
1.00
3.15

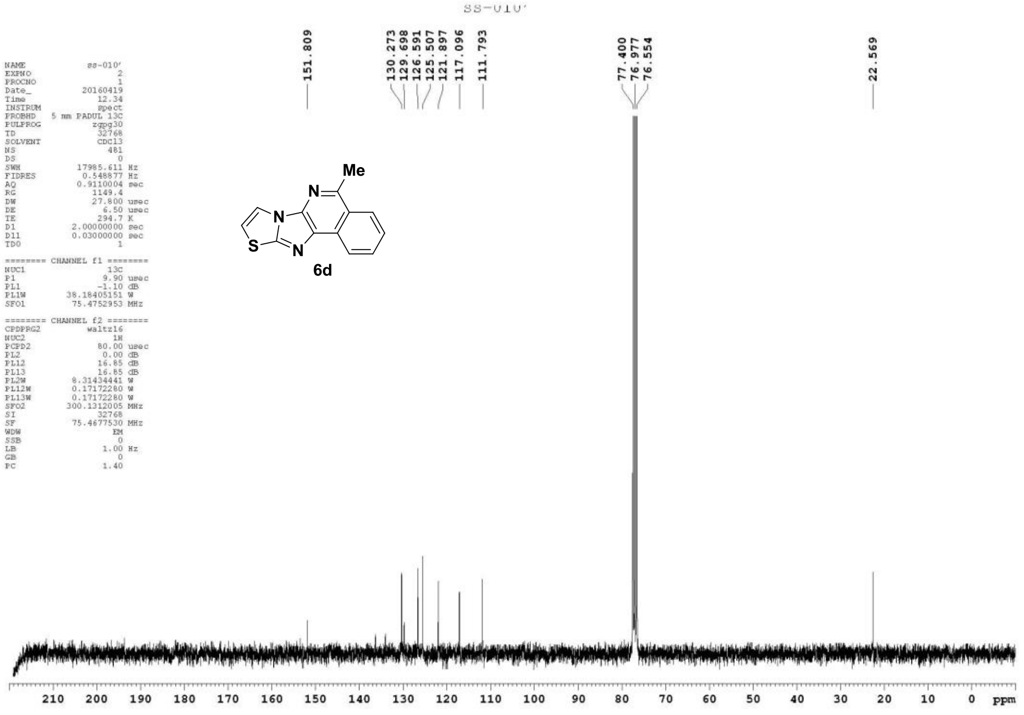
ss-010'
151.809
130.273
129.698
126.591
125.507
121.897
117.096
111.793
77.400
76.977
76.554
22.569
NAME ss-010'
EXPNO 2
PROCNO 1
Date_ 20160419
Time 12.34
INSTRUM spect
PROBHD 5 mm PADUL 13C
PULPROG zgpg30
TD 32768
SOLVENT CDCl3
NS 481
DS 0
SWH 17985.611 Hz
FIDRES 0.548877 Hz
AQ 0.9110004 sec
RG 1149.4
DW 27.800 usec
DE 6.50 usec
TE 294.7 K
D1 2.00000000 sec
D11 0.03000000 sec
TD0 1
======== CHANNEL f1 ========
NUC1 13C
P1 9.90 usec
PL1 -1.10 dB
PL1W 38.18405151 W
SFO1 75.4752953 MHz
======== CHANNEL f2 ========
CPDPRG2 waltz16
NUC2 1H
PCPD2 80.00 usec
PL2 0.00 dB
PL12 16.85 dB
PL13 16.85 dB
PL2W 8.31434441 W
PL12W 0.17172280 W
PL13W 0.17172280 W
SFO2 300.1312005 MHz
SI 32768
SF 75.4677530 MHz
WDW EM
SSB 0
LB 1.00 Hz
GB 0
PC 1.40
Me
N
N
S
N
6d
210
200
190
180
170
160
150
140
130
120
110
100
90
80
70
60
50
40
30
20
10
0
ppm

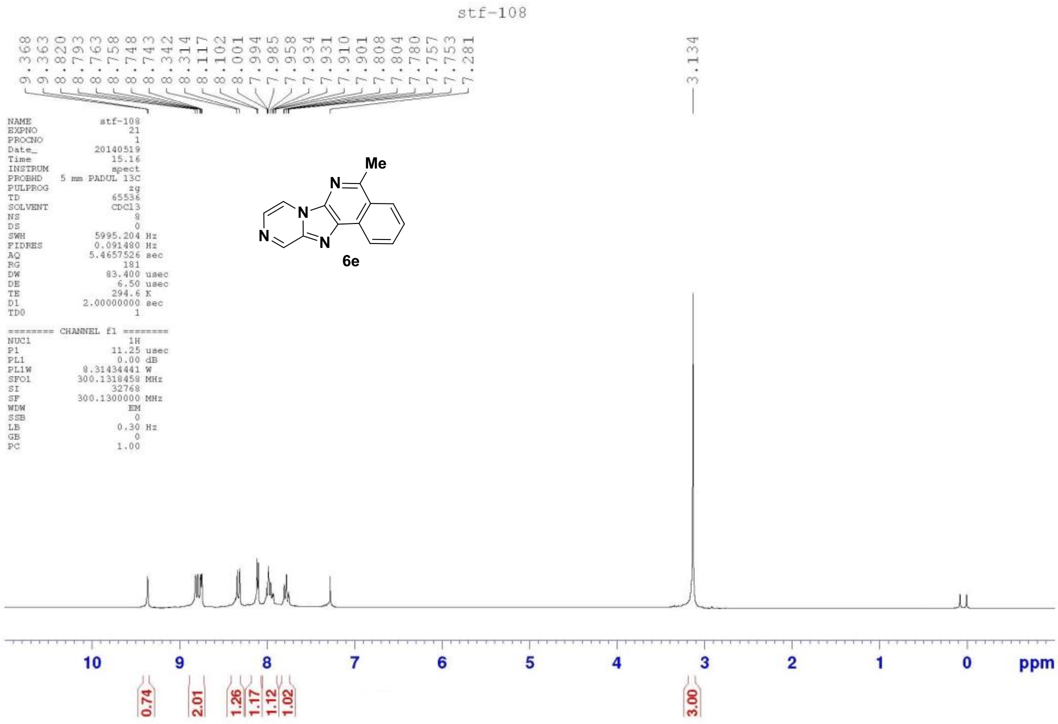
stf-108
9.368
9.363
8.820
8.793
8.763
8.758
8.748
8.743
8.342
8.314
8.117
8.102
8.001
7.994
7.985
7.958
7.934
7.931
7.910
7.901
7.808
7.804
7.780
7.757
7.753
7.281
3.134
NAME stf-108
EXPNO 21
PROCNO 1
Date_ 20140519
Time 15.16
INSTRUM spect
PROBHD 5 mm PADUL 13C
PULPROG zg
TD 65536
SOLVENT CDCl3
NS 8
DS 0
SWH 5995.204 Hz
FIDRES 0.091480 Hz
AQ 5.4657526 sec
RG 181
DW 83.400 usec
DE 6.50 usec
TE 294.6 K
D1 2.00000000 sec
TD0 1
======== CHANNEL f1 ========
NUC1 1H
P1 11.25 usec
PL1 0.00 dB
PL1W 8.31434441 W
SFO1 300.1318458 MHz
SI 32768
SF 300.1300000 MHz
WDW EM
SSB 0
LB 0.30 Hz
GB 0
PC 1.00
Me
N
N
N
N
6e
10
9
8
7
6
5
4
3
2
1
0
ppm
0.74
2.01
1.26
1.17
1.12
1.02
3.00

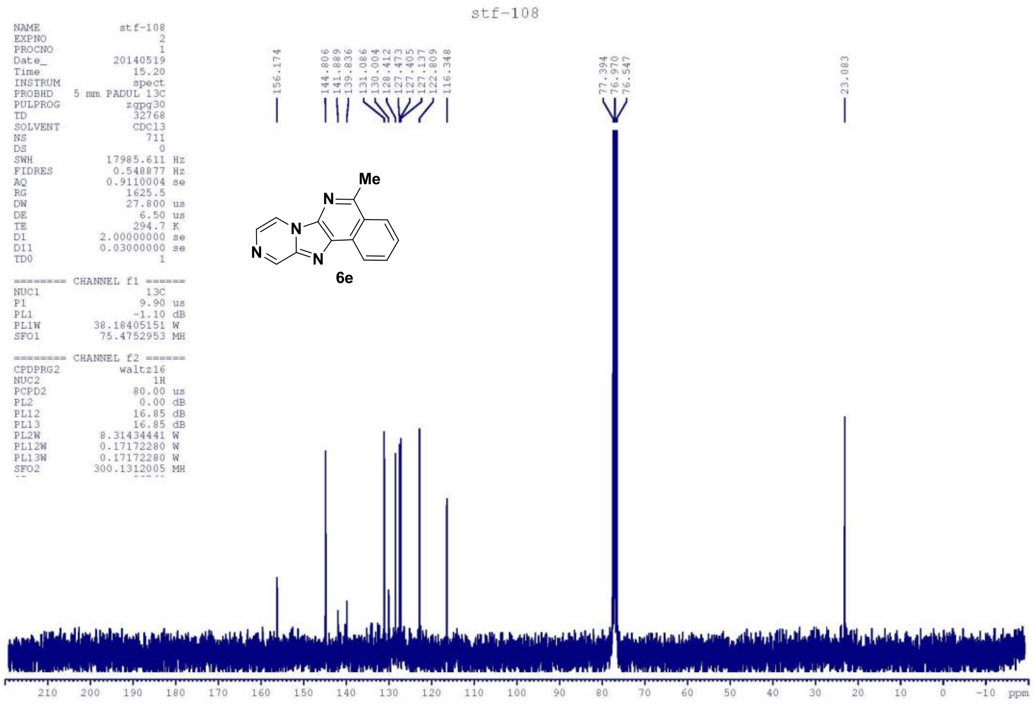
stf-108
NAME stf-108
EXPNO 2
PROCNO 1
Date_ 20140519
Time 15.20
INSTRUM spect
PROBHD 5 mm PADUL 13C
PULPROG zgpg30
TD 32768
SOLVENT CDCl3
NS 711
DS 0
SWH 17985.611 Hz
FIDRES 0.548877 Hz
AQ 0.9110004 se
RG 1625.5
DW 27.800 us
DE 6.50 us
TE 294.7 K
D1 2.00000000 se
D11 0.03000000 se
TD0 1
======== CHANNEL f1 ======
NUC1 13C
P1 9.90 us
PL1 -1.10 dB
PL1W 38.18405151 W
SFO1 75.4752953 MH
======== CHANNEL f2 ======
CPDPRG2 waltz16
NUC2 1H
PCPD2 80.00 us
PL2 0.00 dB
PL12 16.85 dB
PL13 16.85 dB
PL2W 8.31434441 W
PL12W 0.17172280 W
PL13W 0.17172280 W
SFO2 300.1312005 MH
156.174
144.806
141.889
139.836
131.086
130.004
128.412
127.473
127.405
127.137
122.809
116.348
77.394
76.970
76.547
23.083
Me
N
N
N
N
6e
210
200
190
180
170
160
150
140
130
120
110
100
90
80
70
60
50
40
30
20
10
0
-10
ppm

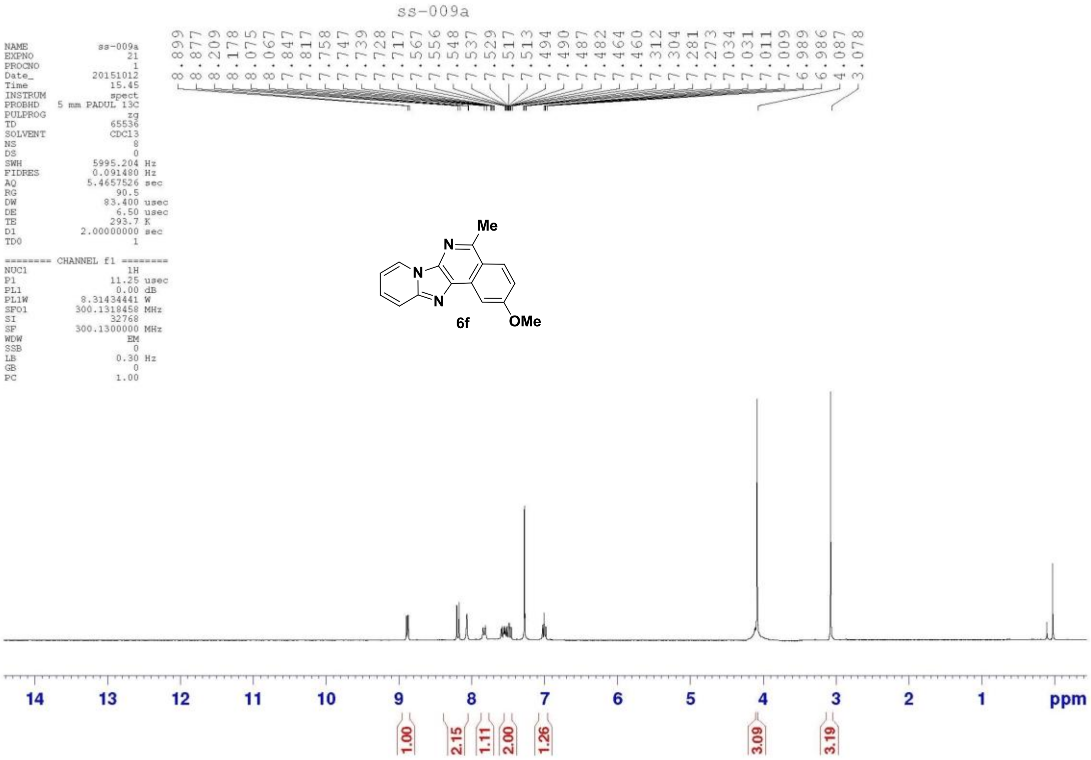
ss-009a
NAME ss-009a
EXPNO 21
PROCNO 1
Date_ 20151012
Time 15.45
INSTRUM spect
PROBHD 5 mm PADUL 13C
PULPROG zg
TD 65536
SOLVENT CDCl3
NS 8
DS 0
SWH 5995.204 Hz
FIDRES 0.091480 Hz
AQ 5.4657526 sec
RG 90.5
DW 83.400 usec
DE 6.50 usec
TE 293.7 K
D1 2.00000000 sec
TD0 1
======== CHANNEL f1 ========
NUC1 1H
P1 11.25 usec
PL1 0.00 dB
PL1W 8.31434441 W
SFO1 300.1318458 MHz
SI 32768
SF 300.1300000 MHz
WDW EM
SSB 0
LB 0.30 Hz
GB 0
PC 1.00
8.899 8.877 8.209 8.178 8.075 8.067 7.847 7.817 7.758 7.747 7.739 7.728 7.717 7.567 7.556 7.548 7.537 7.529 7.517 7.513 7.494 7.490 7.487 7.482 7.464 7.460 7.312 7.304 7.281 7.273 7.034 7.031 7.011 7.009 6.989 6.986 4.087 3.078
Me
N
N
N
6f
OMe
14 13 12 11 10 9 8 7 6 5 4 3 2 1 ppm
1.00 2.15 1.11 2.00 1.26 3.09 3.19

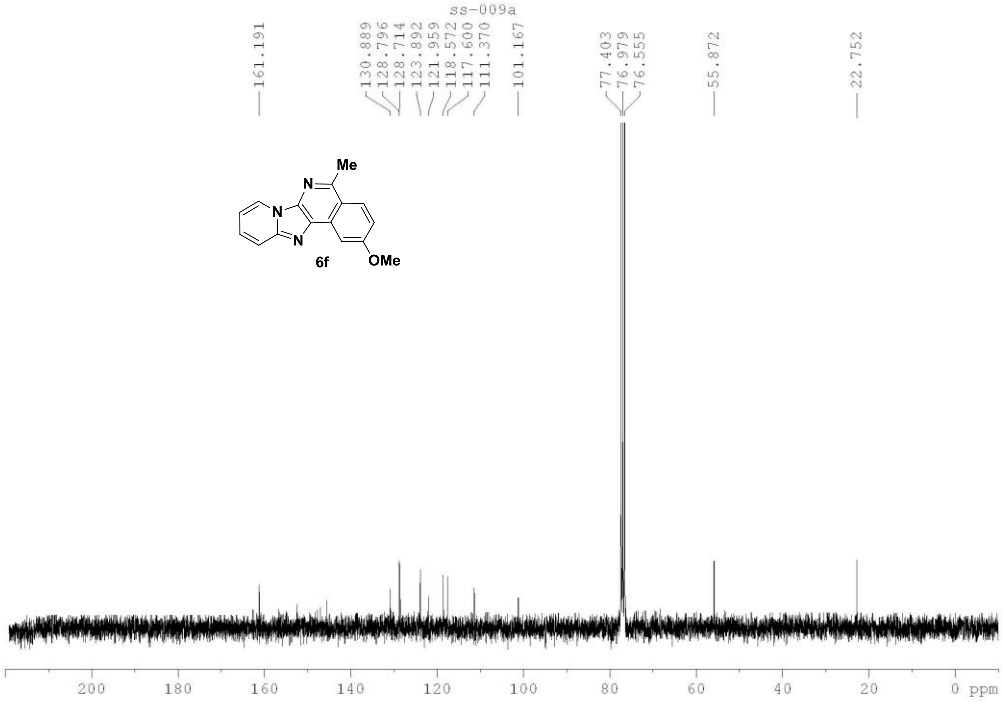
ss-009a
161.191
130.889 128.796 128.714 123.892 121.959 118.572 117.600 111.370
101.167
77.403 76.979 76.555
55.872
22.752
Me
N
N
N
6f
OMe
200 180 160 140 120 100 80 60 40 20 0 ppm

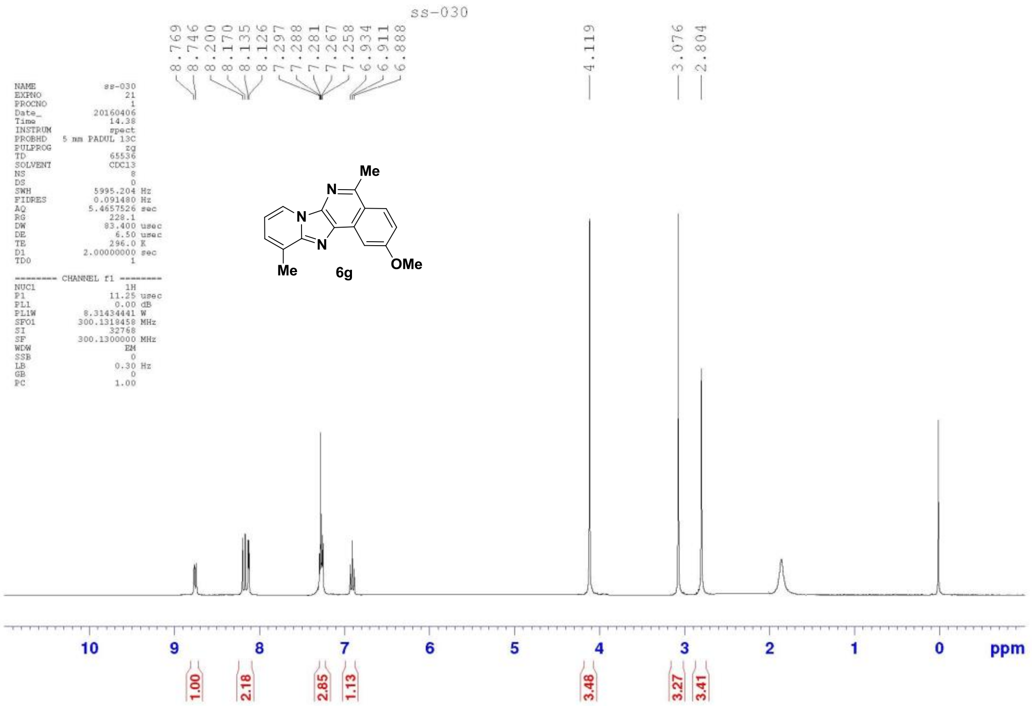
ss-030
8.769
8.746
8.200
8.170
8.135
8.126
7.297
7.288
7.281
7.267
7.258
6.934
6.911
6.888
4.119
3.076
2.804
NAME ss-030
EXPNO 21
PROCNO 1
Date_ 20160406
Time 14.38
INSTRUM spect
PROBHD 5 mm PADUL 13C
PULPROG zg
TD 65536
SOLVENT CDCl3
NS 8
DS 0
SWH 5995.204 Hz
FIDRES 0.091480 Hz
AQ 5.4657526 sec
RG 228.1
DW 83.400 usec
DE 6.50 usec
TE 296.0 K
D1 2.00000000 sec
TD0 1
======== CHANNEL f1 ========
NUC1 1H
P1 11.25 usec
PL1 0.00 dB
PL1W 8.31434441 W
SFO1 300.1318458 MHz
SI 32768
SF 300.1300000 MHz
WDW EM
SSB 0
LB 0.30 Hz
GB 0
PC 1.00
Me
N
N
N
Me
OMe
6g
10
9
8
7
6
5
4
3
2
1
0
ppm
1.00
2.18
2.85
1.13
3.48
3.27
3.41

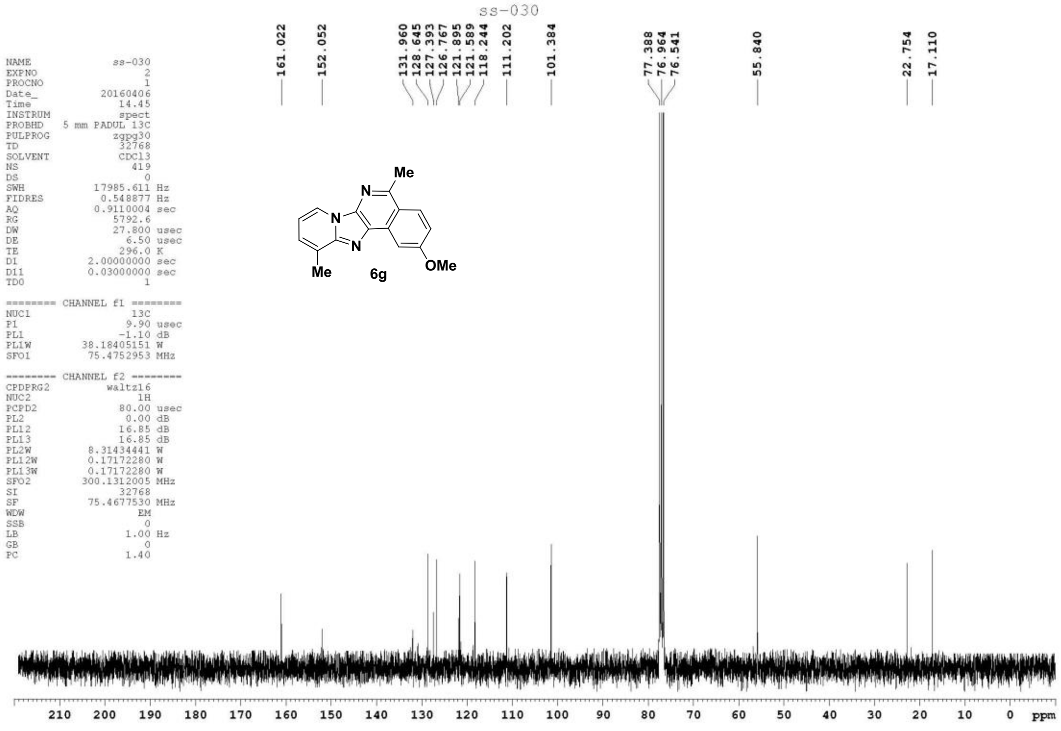
ss-030
161.022
152.052
131.960
128.645
127.393
126.767
121.895
121.589
118.244
111.202
101.384
77.388
76.964
76.541
55.840
22.754
17.110
NAME ss-030
EXPNO 2
PROCNO 1
Date_ 20160406
Time 14.45
INSTRUM spect
PROBHD 5 mm PADUL 13C
PULPROG zgpg30
TD 32768
SOLVENT CDCl3
NS 419
DS 0
SWH 17985.611 Hz
FIDRES 0.548877 Hz
AQ 0.9110004 sec
RG 5792.6
DW 27.800 usec
DE 6.50 usec
TE 296.0 K
D1 2.00000000 sec
D11 0.03000000 sec
TD0 1
======== CHANNEL f1 ========
NUC1 13C
P1 9.90 usec
PL1 -1.10 dB
PL1W 38.18405151 W
SFO1 75.4752953 MHz
======== CHANNEL f2 ========
CPDPRG2 waltz16
NUC2 1H
PCPD2 80.00 usec
PL2 0.00 dB
PL12 16.85 dB
PL13 16.85 dB
PL2W 8.31434441 W
PL12W 0.17172280 W
PL13W 0.17172280 W
SFO2 300.1312005 MHz
SI 32768
SF 75.4677530 MHz
WDW EM
SSB 0
LB 1.00 Hz
GB 0
PC 1.40
Me
N
N
N
Me
OMe
6g
210
200
190
180
170
160
150
140
130
120
110
100
90
80
70
60
50
40
30
20
10
0
ppm

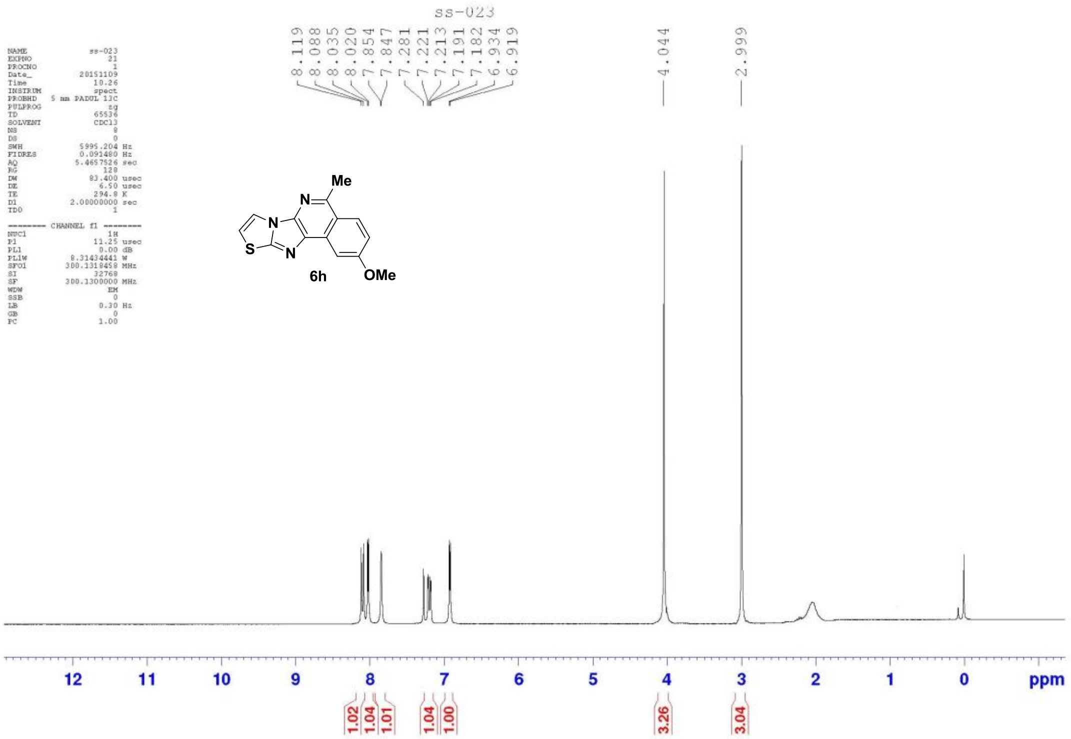
ss-023
8.119
8.088
8.035
8.020
7.854
7.847
7.281
7.221
7.213
7.191
7.182
6.934
6.919
4.044
2.999
Me
N
N
S
N
6h
OMe
12 11 10 9 8 7 6 5 4 3 2 1 0 ppm
1.02
1.04
1.01
1.04
1.00
3.26
3.04

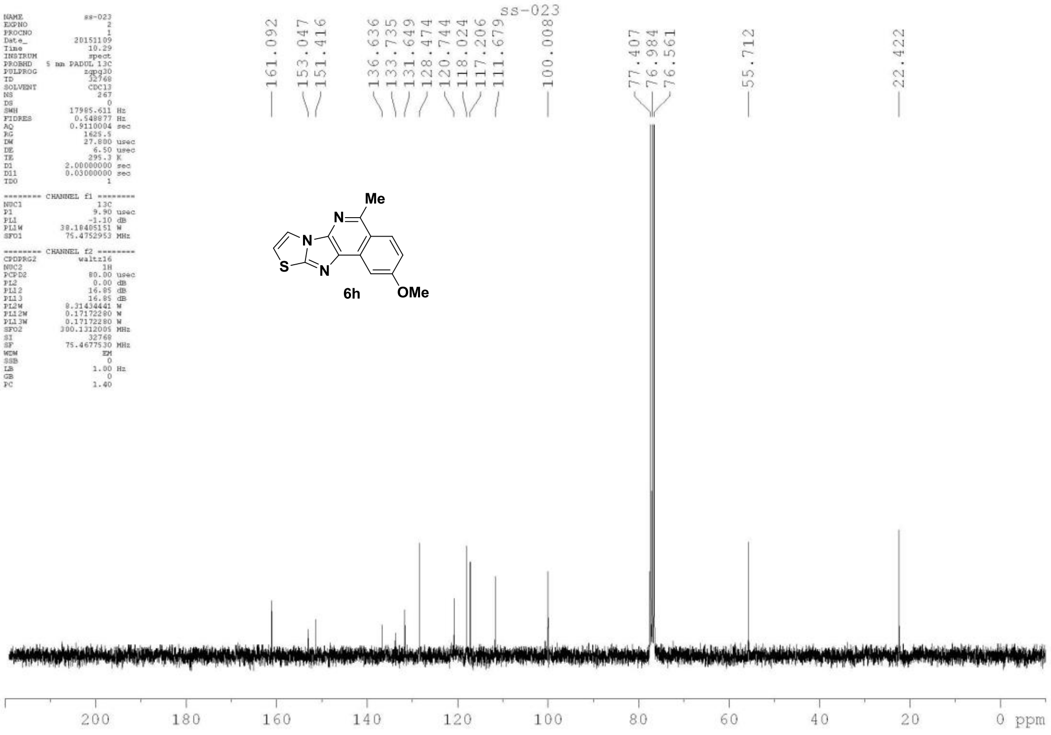
ss-023
161.092
153.047
151.416
136.636
133.735
131.649
128.474
120.744
118.024
117.206
111.679
100.008
77.407
76.984
76.561
55.712
22.422
Me
N
N
S
N
6h
OMe
200 180 160 140 120 100 80 60 40 20 0 ppm

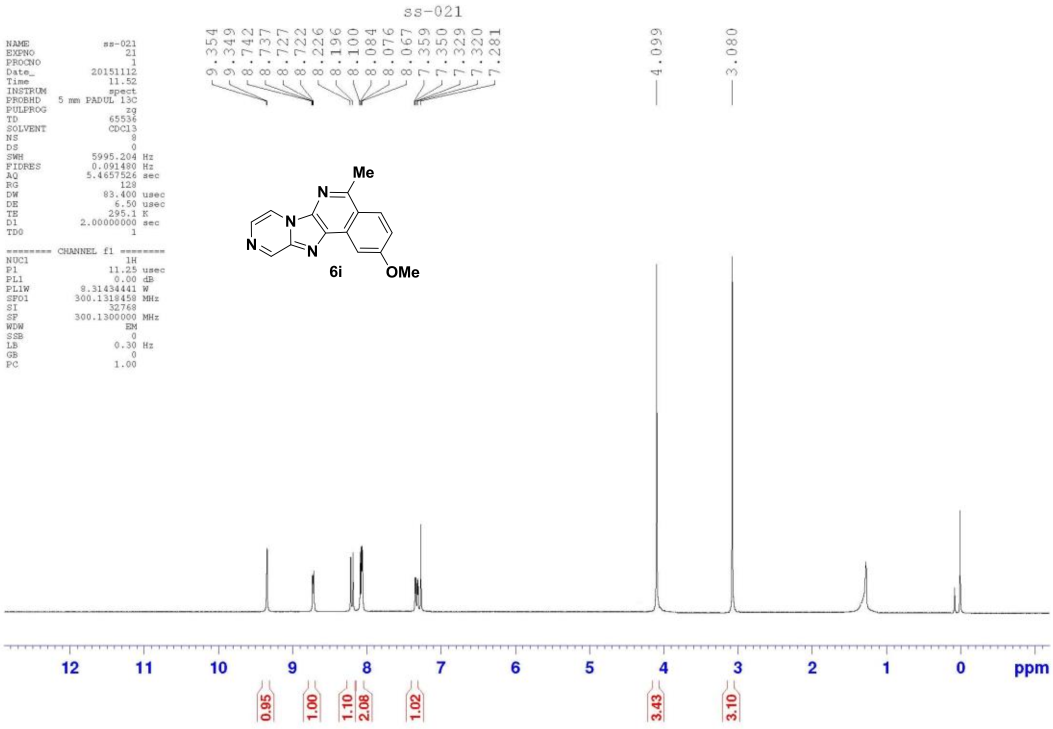
ss-021
NAME ss-021
EXPNO 21
PROCNO 1
Date_ 20151112
Time 11.52
INSTRUM spect
PROBHD 5 mm PADUL 13C
PULPROG zg
TD 65536
SOLVENT CDCl3
NS 8
DS 0
SWH 5995.204 Hz
FIDRES 0.091480 Hz
AQ 5.4657526 sec
RG 128
DW 83.400 usec
DE 6.50 usec
TE 295.1 K
D1 2.00000000 sec
TD0 1
======== CHANNEL f1 ========
NUC1 1H
P1 11.25 usec
PL1 0.00 dB
PL1W 8.31434441 W
SFO1 300.1318459 MHz
SI 32768
SF 300.1300000 MHz
WDW EM
SSB 0
LB 0.30 Hz
GB 0
PC 1.00
9.354
9.349
8.742
8.737
8.727
8.722
8.226
8.196
8.100
8.084
8.076
8.067
7.359
7.350
7.329
7.320
7.281
4.099
3.080
Me
N
N
N
N
6i
OMe
12 11 10 9 8 7 6 5 4 3 2 1 0 ppm
0.95
1.00
1.10
2.08
1.02
3.43
3.10

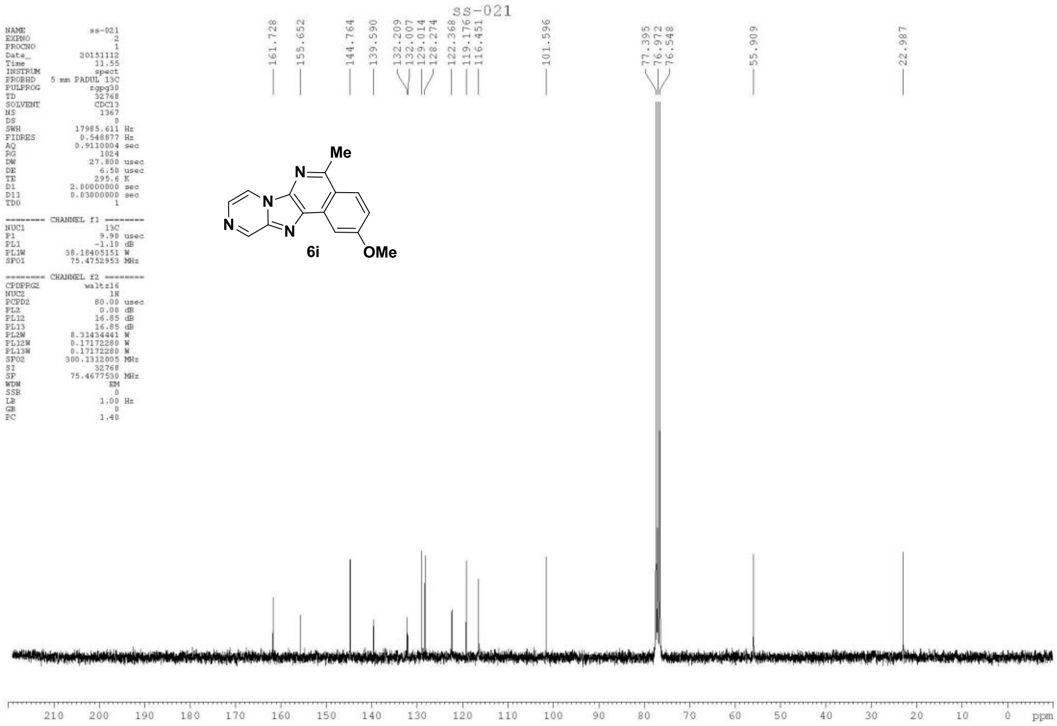
ss-021
NAME ss-021
EXPNO 2
PROCNO 1
Date_ 20151112
Time 11.55
INSTRUM spect
PROBHD 5 mm PADUL 13C
PULPROG zgpg30
TD 32768
SOLVENT CDCl3
NS 1367
DS 0
SWH 17985.611 Hz
FIDRES 0.548877 Hz
AQ 0.9110004 sec
RG 1024
DW 27.800 usec
DE 6.50 usec
TE 295.6 K
D1 2.00000000 sec
D11 0.03000000 sec
TD0 1
======== CHANNEL f1 ========
NUC1 13C
P1 9.90 usec
PL1 -1.10 dB
PL1W 38.18405151 W
SFO1 75.4752953 MHz
======== CHANNEL f2 ========
CPDPRG2 waltz16
NUC2 1H
PCPD2 80.00 usec
PL2 0.00 dB
PL12 16.85 dB
PL13 16.85 dB
PL2W 8.31434441 W
PL12W 0.17172280 W
PL13W 0.17172280 W
SFO2 300.1312005 MHz
SI 32768
SF 75.4677530 MHz
WDW EM
SSB 0
LB 1.00 Hz
GB 0
PC 1.40
161.728
155.652
144.764
139.590
132.209
132.007
129.014
128.274
122.368
119.176
116.451
101.596
77.395
76.972
76.548
55.909
22.987
Me
N
N
N
N
6i
OMe
210 200 190 180 170 160 150 140 130 120 110 100 90 80 70 60 50 40 30 20 10 0 ppm

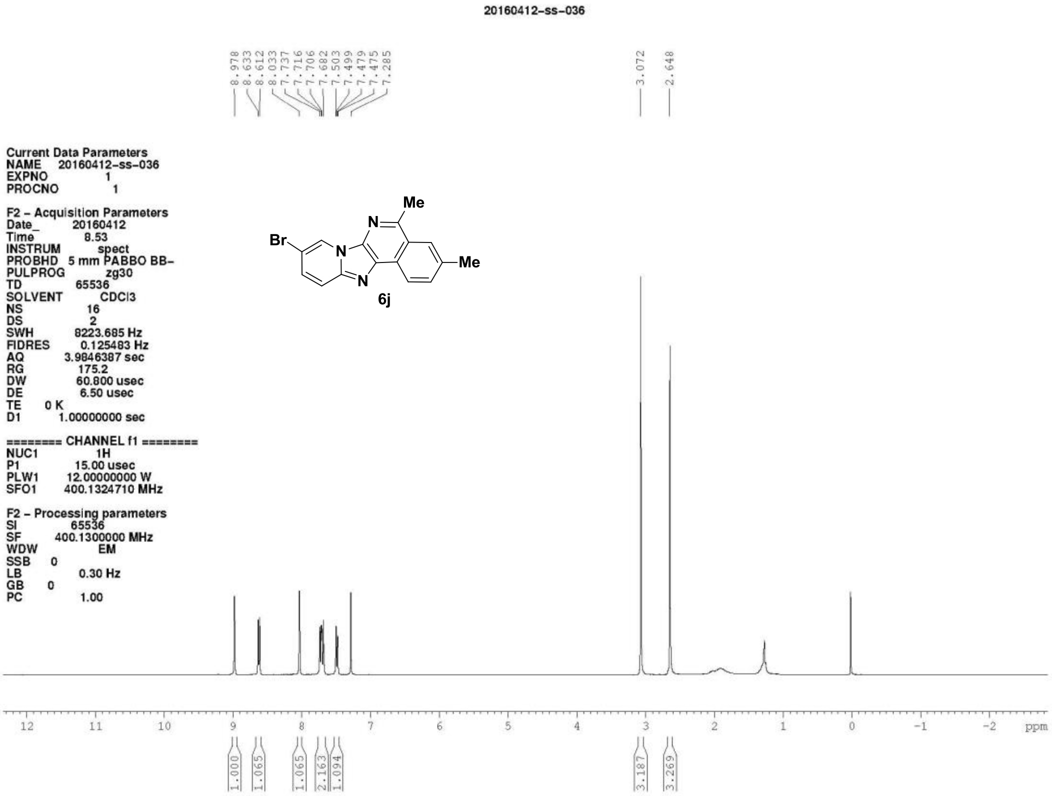
20160412-ss-036
8.978
8.633
8.612
8.033
7.737
7.716
7.706
7.682
7.503
7.499
7.479
7.475
7.285
3.072
2.648
Current Data Parameters
NAME 20160412-ss-036
EXPNO 1
PROCNO 1
F2 - Acquisition Parameters
Date_ 20160412
Time 8.53
INSTRUM spect
PROBHD 5 mm PABBO BB-
PULPROG zg30
TD 65536
SOLVENT CDCl3
NS 16
DS 2
SWH 8223.685 Hz
FIDRES 0.125483 Hz
AQ 3.9846387 sec
RG 175.2
DW 60.800 usec
DE 6.50 usec
TE 0 K
D1 1.00000000 sec
======== CHANNEL f1 ========
NUC1 1H
P1 15.00 usec
PLW1 12.00000000 W
SFO1 400.1324710 MHz
F2 - Processing parameters
SI 65536
SF 400.1300000 MHz
WDW EM
SSB 0
LB 0.30 Hz
GB 0
PC 1.00
Me
N
Br
N
N
Me
6j
12
11
10
9
8
7
6
5
4
3
2
1
0
-1
-2
ppm
1.000
1.065
1.065
2.163
1.094
3.187
3.269

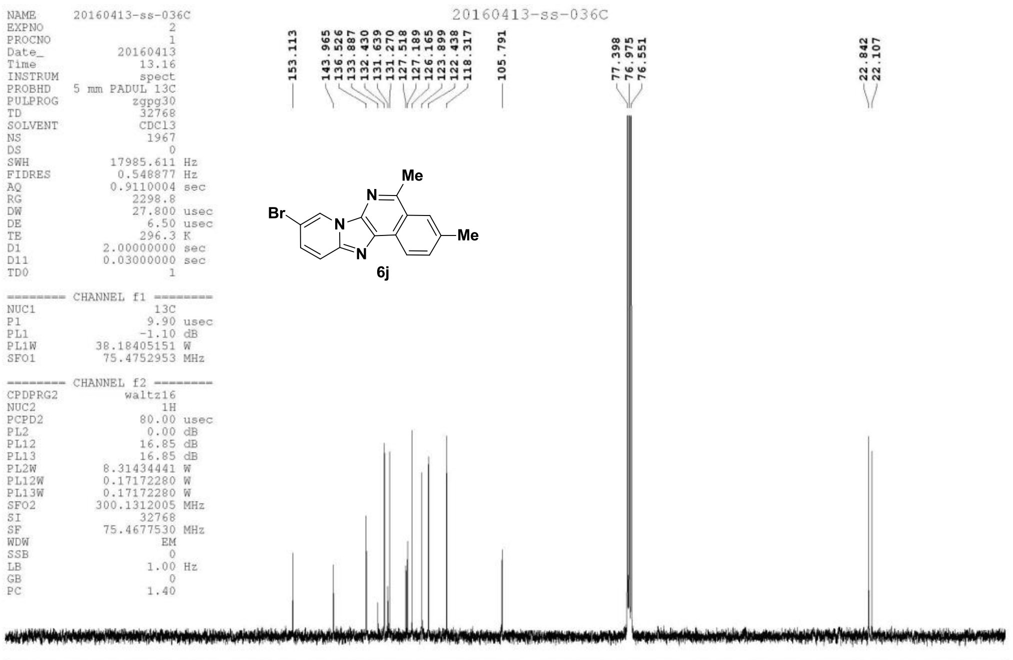
NAME 20160413-ss-036C
EXPNO 2
PROCNO 1
Date_ 20160413
Time 13.16
INSTRUM spect
PROBHD 5 mm PADUL 13C
PULPROG zgpg30
TD 32768
SOLVENT CDCl3
NS 1967
DS 0
SWH 17985.611 Hz
FIDRES 0.548877 Hz
AQ 0.9110004 sec
RG 2298.8
DW 27.800 usec
DE 6.50 usec
TE 296.3 K
D1 2.00000000 sec
D11 0.03000000 sec
TD0 1
======== CHANNEL f1 ========
NUC1 13C
P1 9.90 usec
PL1 -1.10 dB
PL1W 38.18405151 W
SFO1 75.4752953 MHz
======== CHANNEL f2 ========
CPDPRG2 waltz16
NUC2 1H
PCPD2 80.00 usec
PL2 0.00 dB
PL12 16.85 dB
PL13 16.85 dB
PL2W 8.31434441 W
PL12W 0.17172280 W
PL13W 0.17172280 W
SFO2 300.1312005 MHz
SI 32768
SF 75.4677530 MHz
WDW EM
SSB 0
LB 1.00 Hz
GB 0
PC 1.40
20160413-ss-036C
153.113
143.965
136.526
133.887
132.430
131.639
131.270
127.518
127.189
126.165
123.899
122.438
118.317
105.791
77.398
76.975
76.551
22.842
22.107
Me
N
Br
N
N
Me
6j
210
200
190
180
170
160
150
140
130
120
110
100
90
80
70
60
50
40
30
20
10
0
ppm

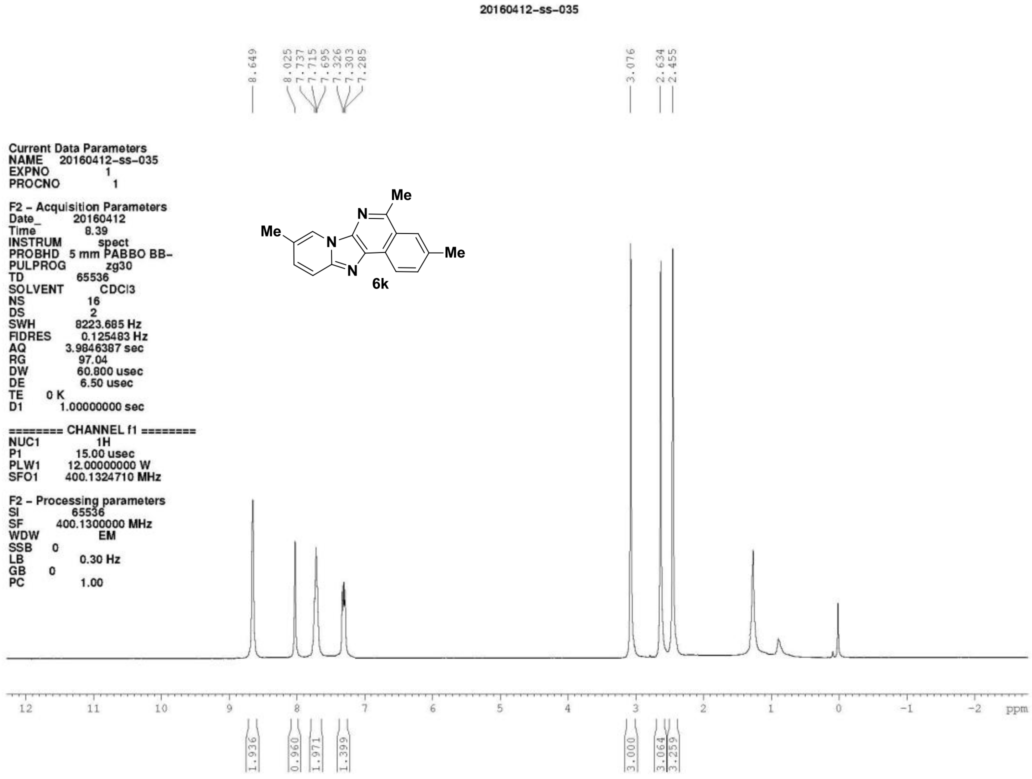
20160412-ss-035
8.649
8.025
7.737
7.715
7.695
7.326
7.303
7.285
3.076
2.634
2.455
Current Data Parameters
NAME 20160412-ss-035
EXPNO 1
PROCNO 1
F2 - Acquisition Parameters
Date_ 20160412
Time 8.39
INSTRUM spect
PROBHD 5 mm PABBO BB-
PULPROG zg30
TD 65536
SOLVENT CDCl3
NS 16
DS 2
SWH 8223.685 Hz
FIDRES 0.125483 Hz
AQ 3.9846387 sec
RG 97.04
DW 60.800 usec
DE 6.50 usec
TE 0 K
D1 1.00000000 sec
======== CHANNEL f1 ========
NUC1 1H
P1 15.00 usec
PLW1 12.00000000 W
SFO1 400.1324710 MHz
F2 - Processing parameters
SI 65536
SF 400.1300000 MHz
WDW EM
SSB 0
LB 0.30 Hz
GB 0
PC 1.00
Me
N
Me
N
Me
N
6k
12
11
10
9
8
7
6
5
4
3
2
1
0
-1
-2
ppm
1.936
0.960
1.971
1.399
3.000
3.064
3.259

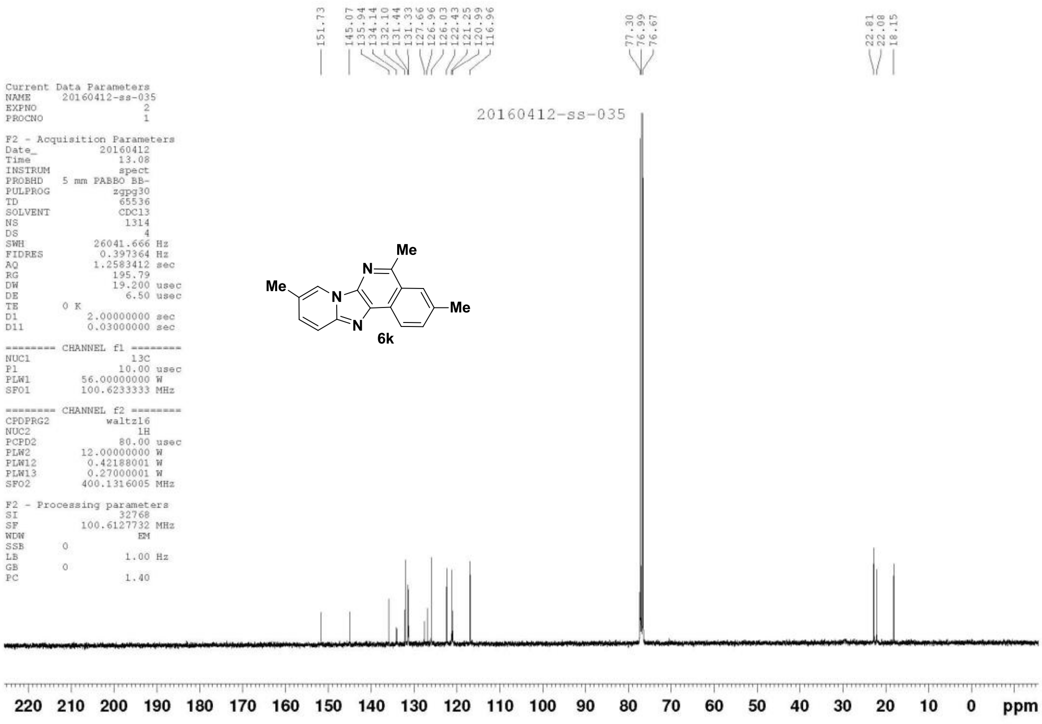
151.73
145.07
135.94
134.14
132.10
131.44
131.33
127.66
126.96
126.03
122.43
121.25
120.99
116.96
77.30
76.99
76.67
22.81
22.08
18.15
Current Data Parameters
NAME 20160412-ss-035
EXPNO 2
PROCNO 1
20160412-ss-035
F2 - Acquisition Parameters
Date_ 20160412
Time 13.08
INSTRUM spect
PROBHD 5 mm PABBO BB-
PULPROG zgpg30
TD 65536
SOLVENT CDCl3
NS 1314
DS 4
SWH 26041.666 Hz
FIDRES 0.397364 Hz
AQ 1.2583412 sec
RG 195.79
DW 19.200 usec
DE 6.50 usec
TE 0 K
D1 2.00000000 sec
D11 0.03000000 sec
======== CHANNEL f1 ========
NUC1 13C
P1 10.00 usec
PLW1 56.00000000 W
SFO1 100.6233333 MHz
======== CHANNEL f2 ========
CPDPRG2 waltz16
NUC2 1H
PCPD2 80.00 usec
PLW2 12.00000000 W
PLW12 0.42188001 W
PLW13 0.27000001 W
SFO2 400.1316005 MHz
F2 - Processing parameters
SI 32768
SF 100.6127732 MHz
WDW EM
SSB 0
LB 1.00 Hz
GB 0
PC 1.40
Me
N
Me
N
Me
N
6k
220
210
200
190
180
170
160
150
140
130
120
110
100
90
80
70
60
50
40
30
20
10
0
ppm

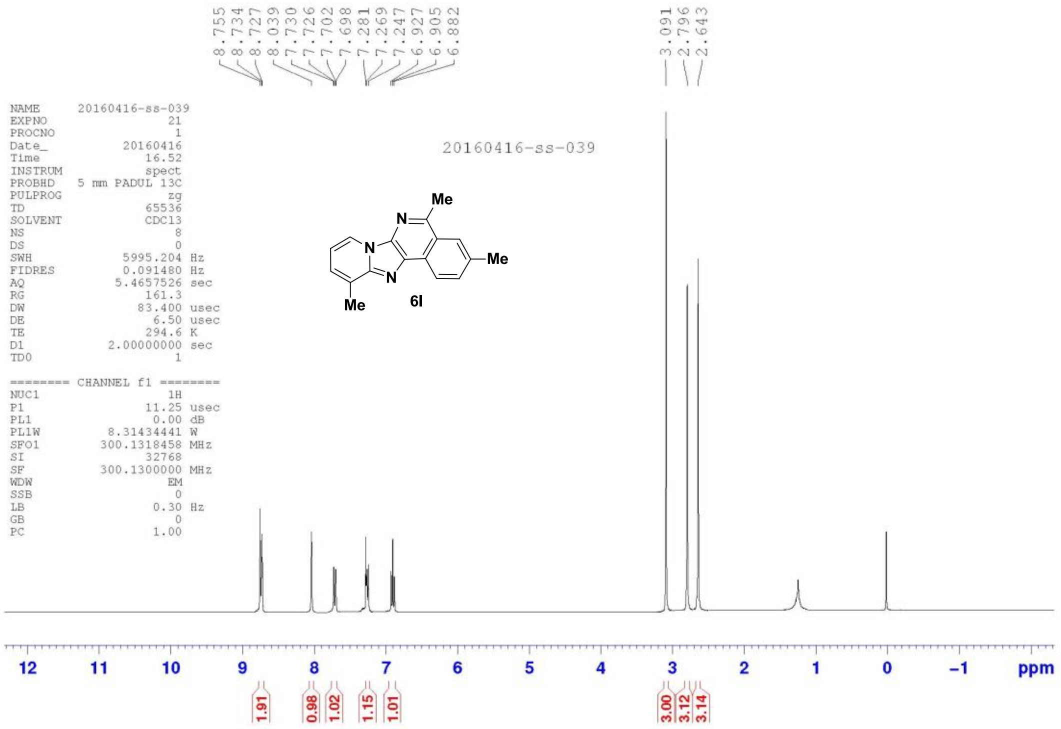
8.755
8.734
8.727
8.039
7.730
7.726
7.702
7.698
7.281
7.269
7.247
6.927
6.905
6.882
3.091
2.796
2.643
NAME 20160416-ss-039
EXPNO 21
PROCNO 1
Date_ 20160416
Time 16.52
INSTRUM spect
PROBHD 5 mm PADUL 13C
PULPROG zg
TD 65536
SOLVENT CDCl3
NS 8
DS 0
SWH 5995.204 Hz
FIDRES 0.091480 Hz
AQ 5.4657526 sec
RG 161.3
DW 83.400 usec
DE 6.50 usec
TE 294.6 K
D1 2.00000000 sec
TD0 1
======== CHANNEL f1 ========
NUC1 1H
P1 11.25 usec
PL1 0.00 dB
PL1W 8.31434441 W
SFO1 300.1318458 MHz
SI 32768
SF 300.1300000 MHz
WDW EM
SSB 0
LB 0.30 Hz
GB 0
PC 1.00
20160416-ss-039
Me
N
N
N
Me
Me
6l
12
11
10
9
8
7
6
5
4
3
2
1
0
–1
ppm
1.91
0.98
1.02
1.15
1.01
3.00
3.12
3.14

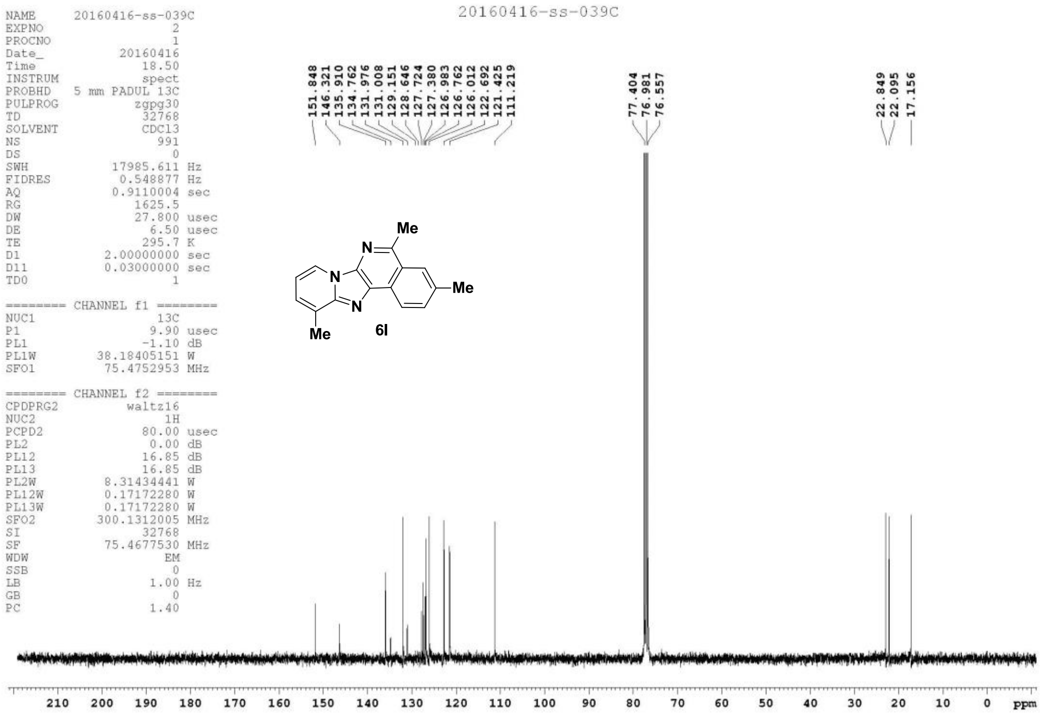
NAME 20160416-ss-039C
EXPNO 2
PROCNO 1
Date_ 20160416
Time 18.50
INSTRUM spect
PROBHD 5 mm PADUL 13C
PULPROG zgpg30
TD 32768
SOLVENT CDCl3
NS 991
DS 0
SWH 17985.611 Hz
FIDRES 0.548877 Hz
AQ 0.9110004 sec
RG 1625.5
DW 27.800 usec
DE 6.50 usec
TE 295.7 K
D1 2.00000000 sec
D11 0.03000000 sec
TD0 1
======== CHANNEL f1 ========
NUC1 13C
P1 9.90 usec
PL1 -1.10 dB
PL1W 38.18405151 W
SFO1 75.4752953 MHz
======== CHANNEL f2 ========
CPDPRG2 waltz16
NUC2 1H
PCPD2 80.00 usec
PL2 0.00 dB
PL12 16.85 dB
PL13 16.85 dB
PL2W 8.31434441 W
PL12W 0.17172280 W
PL13W 0.17172280 W
SFO2 300.1312005 MHz
SI 32768
SF 75.4677530 MHz
WDW EM
SSB 0
LB 1.00 Hz
GB 0
PC 1.40
20160416-ss-039C
151.848
146.321
135.910
134.762
131.976
131.008
129.151
128.646
127.724
127.380
126.983
126.762
126.012
122.692
121.425
111.219
77.404
76.981
76.557
22.849
22.095
17.156
Me
N
N
N
Me
Me
6l
210
200
190
180
170
160
150
140
130
120
110
100
90
80
70
60
50
40
30
20
10
0
ppm

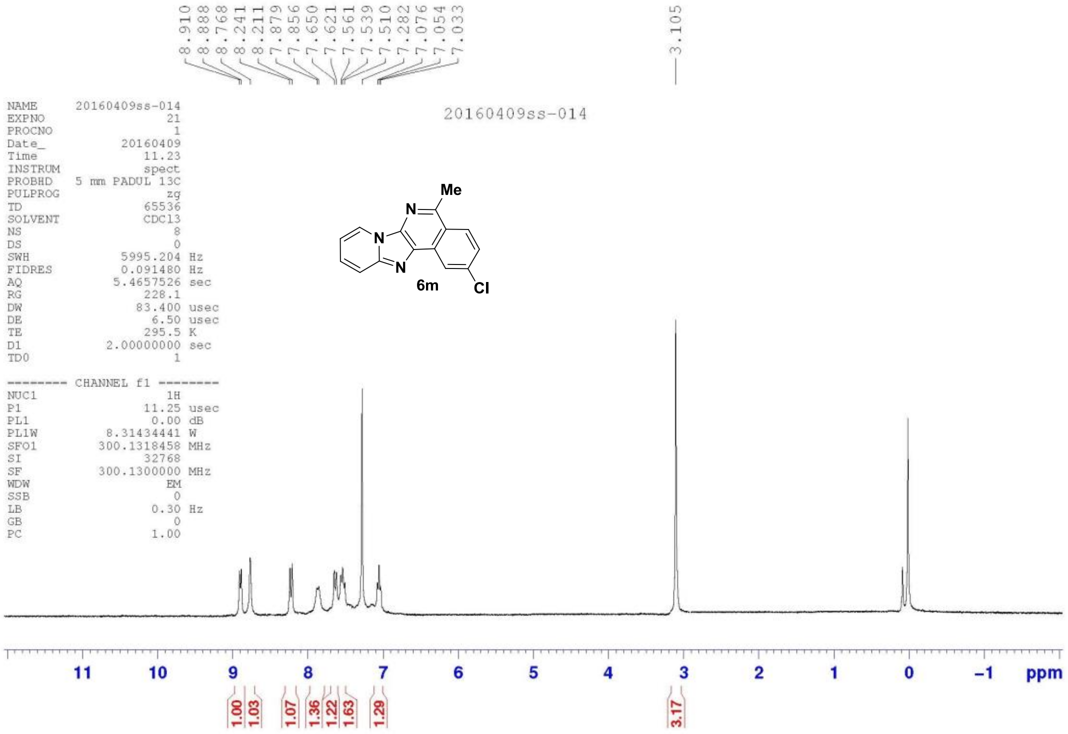
8.910 8.888 8.768 8.241 8.211 7.879 7.856 7.650 7.621 7.561 7.539 7.510 7.282 7.076 7.054 7.033
3.105
20160409ss-014
NAME 20160409ss-014
EXPNO 21
PROCNO 1
Date_ 20160409
Time 11.23
INSTRUM spect
PROBHD 5 mm PADUL 13C
PULPROG zg
TD 65536
SOLVENT CDCl3
NS 8
DS 0
SWH 5995.204 Hz
FIDRES 0.091480 Hz
AQ 5.4657526 sec
RG 228.1
DW 83.400 usec
DE 6.50 usec
TE 295.5 K
D1 2.00000000 sec
TD0 1
======== CHANNEL f1 ========
NUC1 1H
P1 11.25 usec
PL1 0.00 dB
PL1W 8.31434441 W
SFO1 300.1318458 MHz
SI 32768
SF 300.1300000 MHz
WDW EM
SSB 0
LB 0.30 Hz
GB 0
PC 1.00
Me
N
N
N
Cl
6m
11 10 9 8 7 6 5 4 3 2 1 0 −1 ppm
1.00 1.03 1.07 1.36 1.22 1.63 1.29 3.17

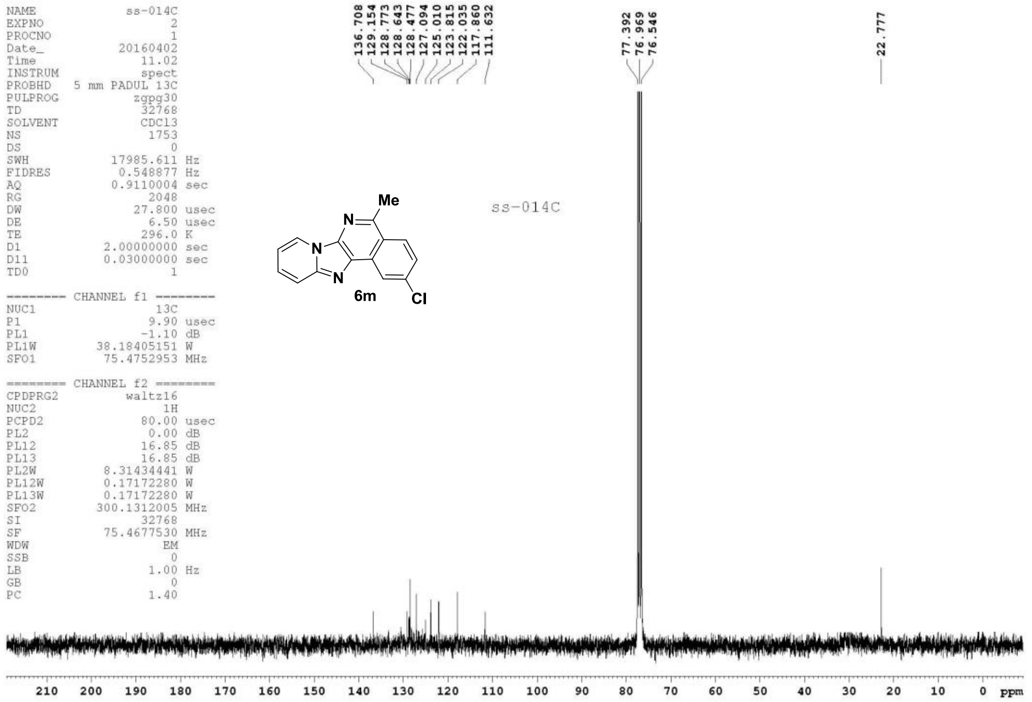
136.708 129.154 128.773 128.643 128.477 127.094 125.010 123.815 122.035 117.860 111.632
77.392 76.969 76.546
22.777
NAME ss-014C
EXPNO 2
PROCNO 1
Date_ 20160402
Time 11.02
INSTRUM spect
PROBHD 5 mm PADUL 13C
PULPROG zgpg30
TD 32768
SOLVENT CDCl3
NS 1753
DS 0
SWH 17985.611 Hz
FIDRES 0.548877 Hz
AQ 0.9110004 sec
RG 2048
DW 27.800 usec
DE 6.50 usec
TE 296.0 K
D1 2.00000000 sec
D11 0.03000000 sec
TD0 1
======== CHANNEL f1 ========
NUC1 13C
P1 9.90 usec
PL1 -1.10 dB
PL1W 38.18405151 W
SFO1 75.4752953 MHz
======== CHANNEL f2 ========
CPDPRG2 waltz16
NUC2 1H
PCPD2 80.00 usec
PL2 0.00 dB
PL12 16.85 dB
PL13 16.85 dB
PL2W 8.31434441 W
PL12W 0.17172280 W
PL13W 0.17172280 W
SFO2 300.1312005 MHz
SI 32768
SF 75.4677530 MHz
WDW EM
SSB 0
LB 1.00 Hz
GB 0
PC 1.40
ss-014C
Me
N
N
N
Cl
6m
210 200 190 180 170 160 150 140 130 120 110 100 90 80 70 60 50 40 30 20 10 0 ppm

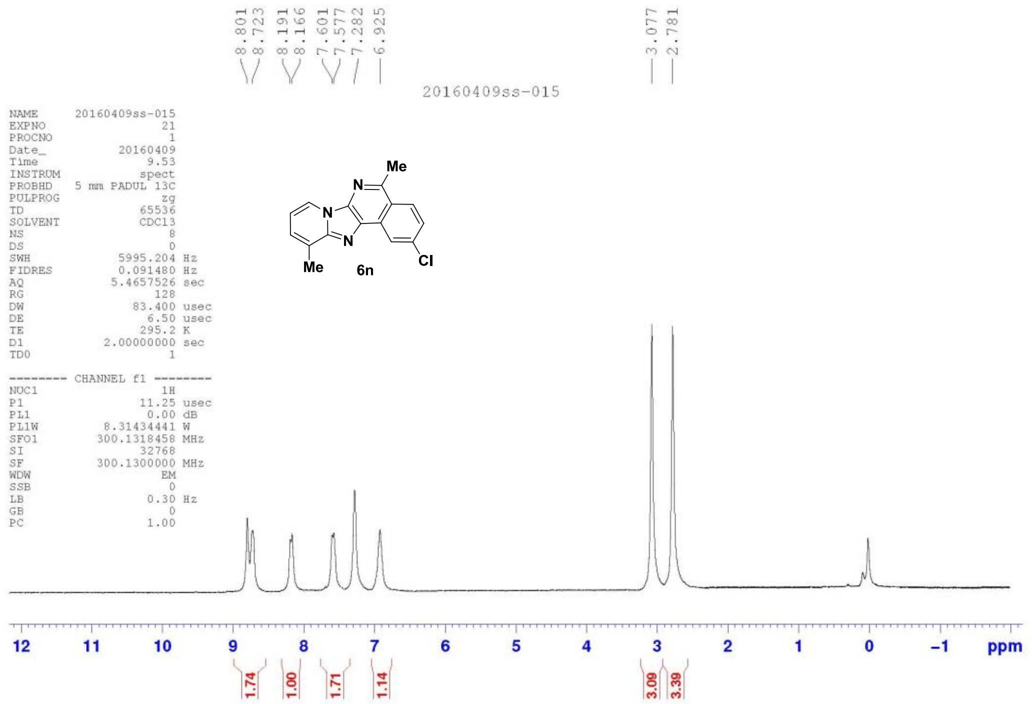
8.801
8.723
8.191
8.166
7.601
7.577
7.282
6.925
3.077
2.781
20160409ss-015
NAME 20160409ss-015
EXPNO 21
PROCNO 1
Date_ 20160409
Time 9.53
INSTRUM spect
PROBHD 5 mm PADUL 13C
PULPROG zg
TD 65536
SOLVENT CDCl3
NS 8
DS 0
SWH 5995.204 Hz
FIDRES 0.091480 Hz
AQ 5.4657526 sec
RG 128
DW 83.400 usec
DE 6.50 usec
TE 295.2 K
D1 2.00000000 sec
TD0 1
======== CHANNEL f1 ========
NUC1 1H
P1 11.25 usec
PL1 0.00 dB
PL1W 8.31434441 W
SFO1 300.1318458 MHz
SI 32768
SF 300.1300000 MHz
WDW EM
SSB 0
LB 0.30 Hz
GB 0
PC 1.00
Me
N
N
N
Me
Cl
6n
12 11 10 9 8 7 6 5 4 3 2 1 0 −1 ppm
1.74
1.00
1.71
1.14
3.09
3.39

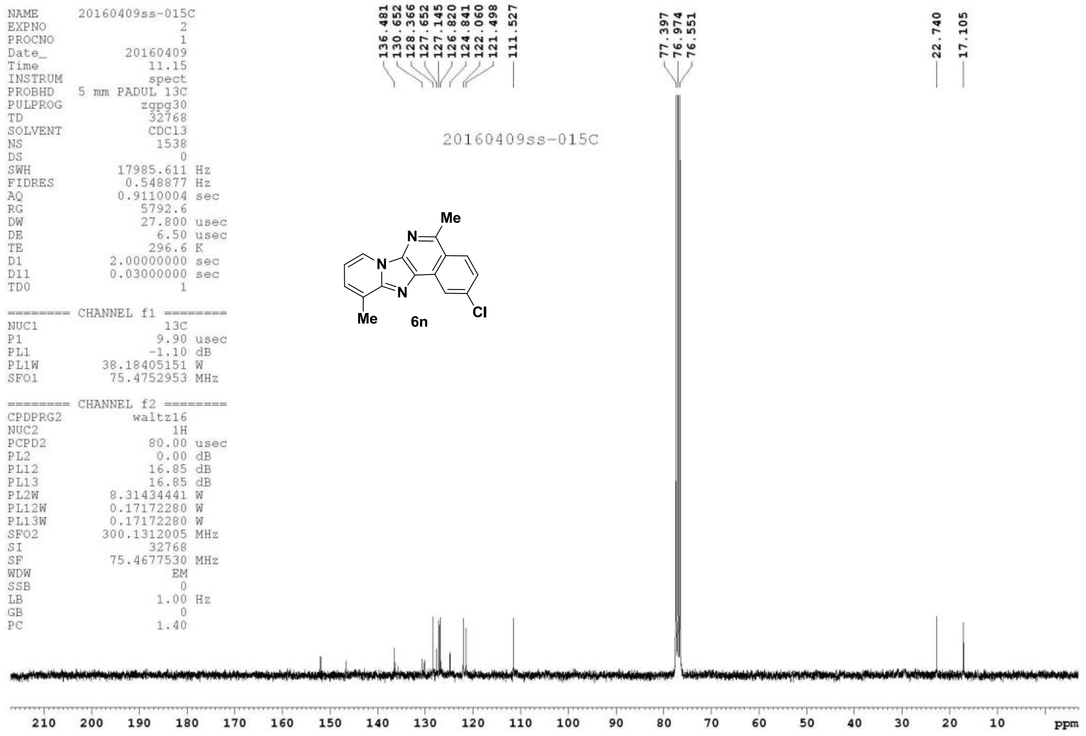
NAME 20160409ss-015C
EXPNO 2
PROCNO 1
Date_ 20160409
Time 11.15
INSTRUM spect
PROBHD 5 mm PADUL 13C
PULPROG zgpg30
TD 32768
SOLVENT CDCl3
NS 1538
DS 0
SWH 17985.611 Hz
FIDRES 0.548877 Hz
AQ 0.9110004 sec
RG 5792.6
DW 27.800 usec
DE 6.50 usec
TE 296.6 K
D1 2.00000000 sec
D11 0.03000000 sec
TD0 1
======== CHANNEL f1 ========
NUC1 13C
P1 9.90 usec
PL1 -1.10 dB
PL1W 38.18405151 W
SFO1 75.4752953 MHz
======== CHANNEL f2 ========
CPDPRG2 waltz16
NUC2 1H
PCPD2 80.00 usec
PL2 0.00 dB
PL12 16.85 dB
PL13 16.85 dB
PL2W 8.31434441 W
PL12W 0.17172280 W
PL13W 0.17172280 W
SFO2 300.1312005 MHz
SI 32768
SF 75.4677530 MHz
WDW EM
SSB 0
LB 1.00 Hz
GB 0
PC 1.40
136.481
130.652
128.366
127.652
127.145
126.820
124.841
122.060
121.498
111.527
77.397
76.974
76.551
22.740
17.105
20160409ss-015C
Me
N
N
N
Me
Cl
6n
210 200 190 180 170 160 150 140 130 120 110 100 90 80 70 60 50 40 30 20 10 ppm

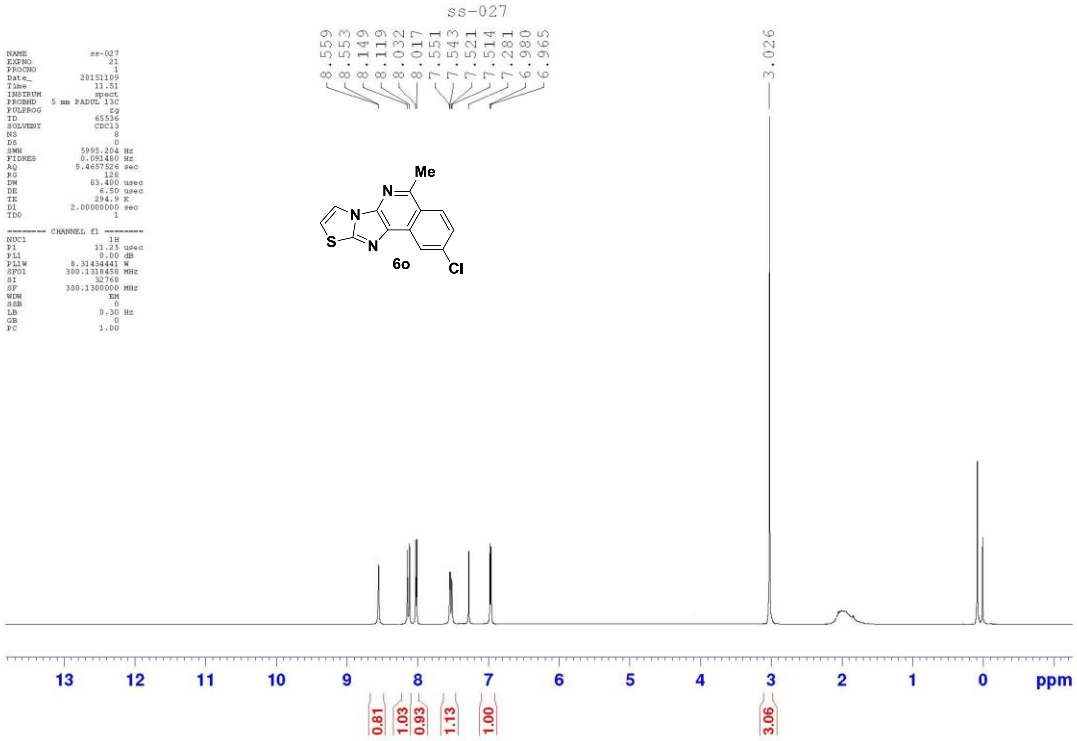
ss-027
8.559
8.553
8.149
8.119
8.032
8.017
7.551
7.543
7.521
7.514
7.281
6.980
6.965
3.026
Me
N
N
S
N
6o
Cl
ppm
0.81
1.03
0.93
1.13
1.00
3.06

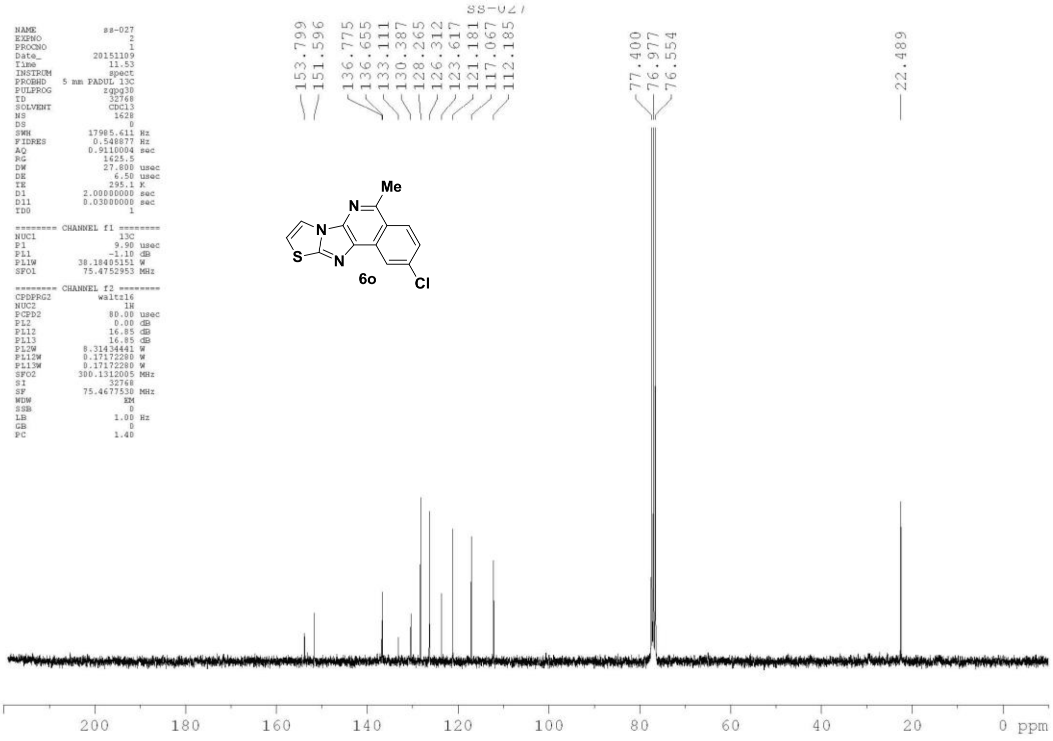
153.799
151.596
136.775
136.655
133.111
130.387
128.265
126.312
123.617
121.181
117.067
112.185
77.400
76.977
76.554
22.489
Me
N
N
S
N
6o
Cl
ppm

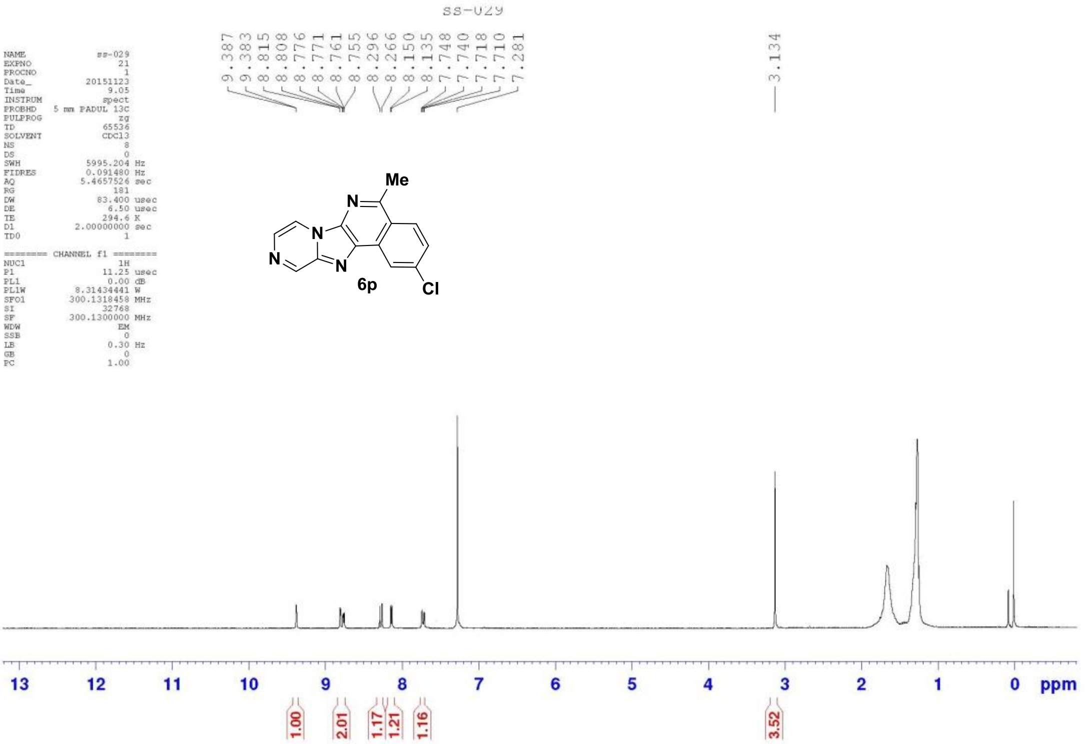
ss-029
9.387
9.383
8.815
8.808
8.776
8.771
8.761
8.755
8.296
8.266
8.150
8.135
7.748
7.740
7.718
7.710
7.281
3.134
NAME ss-029
EXPNO 21
PROCNO 1
Date_ 20151123
Time 9.05
INSTRUM spect
PROBHD 5 mm PADUL 13C
PULPROG zg
TD 65536
SOLVENT CDCl3
NS 8
DS 0
SWH 5995.204 Hz
FIDRES 0.091480 Hz
AQ 5.4657526 sec
RG 181
DW 83.400 usec
DE 6.50 usec
TE 294.6 K
D1 2.00000000 sec
TD0 1
======= CHANNEL f1 =======
NUC1 1H
P1 11.25 usec
PL1 0.00 dB
PL1W 8.31434441 W
SFO1 300.1318458 MHz
SI 32768
SF 300.1300000 MHz
WDW EM
SSB 0
LB 0.30 Hz
GB 0
PC 1.00
Me
N
N
N
N
6p
Cl
13
12
11
10
9
8
7
6
5
4
3
2
1
0
ppm
1.00
2.01
1.17
1.21
1.16
3.52

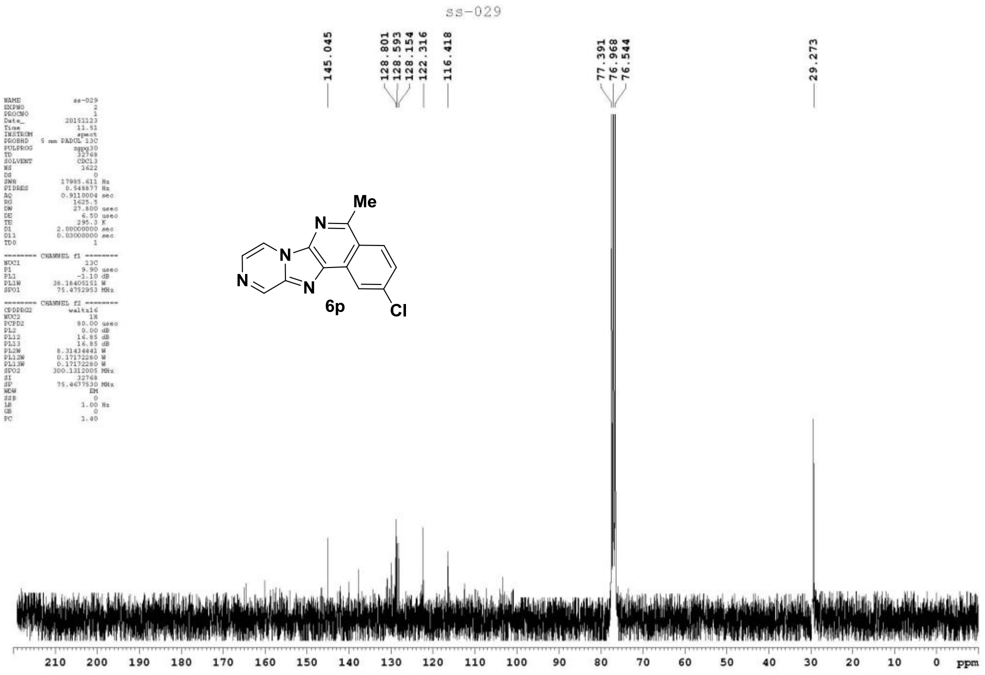
ss-029
145.045
128.801
128.593
128.154
122.316
116.418
77.391
76.968
76.544
29.273
Me
N
N
N
N
6p
Cl
210
200
190
180
170
160
150
140
130
120
110
100
90
80
70
60
50
40
30
20
10
0
ppm

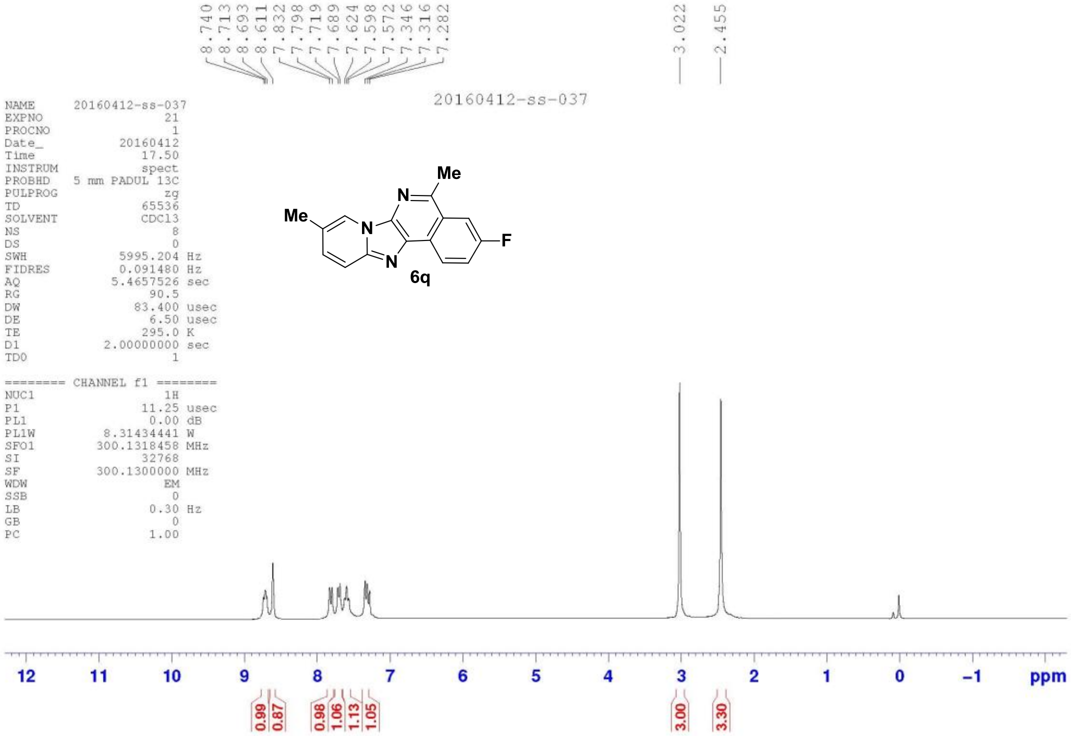
8.740
8.713
8.693
8.611
7.832
7.798
7.719
7.689
7.624
7.598
7.572
7.346
7.316
7.282
3.022
2.455
20160412-ss-037
NAME 20160412-ss-037
EXPNO 21
PROCNO 1
Date_ 20160412
Time 17.50
INSTRUM spect
PROBHD 5 mm PADUL 13C
PULPROG zg
TD 65536
SOLVENT CDCl3
NS 8
DS 0
SWH 5995.204 Hz
FIDRES 0.091480 Hz
AQ 5.4657526 sec
RG 90.5
DW 83.400 usec
DE 6.50 usec
TE 295.0 K
D1 2.00000000 sec
TD0 1
======== CHANNEL f1 ========
NUC1 1H
P1 11.25 usec
PL1 0.00 dB
PL1W 8.31434441 W
SFO1 300.1318458 MHz
SI 32768
SF 300.1300000 MHz
WDW EM
SSB 0
LB 0.30 Hz
GB 0
PC 1.00
Me
N
Me
N
N
F
6q
12
11
10
9
8
7
6
5
4
3
2
1
0
–1
ppm
0.99
0.87
0.98
1.06
1.13
1.05
3.00
3.30

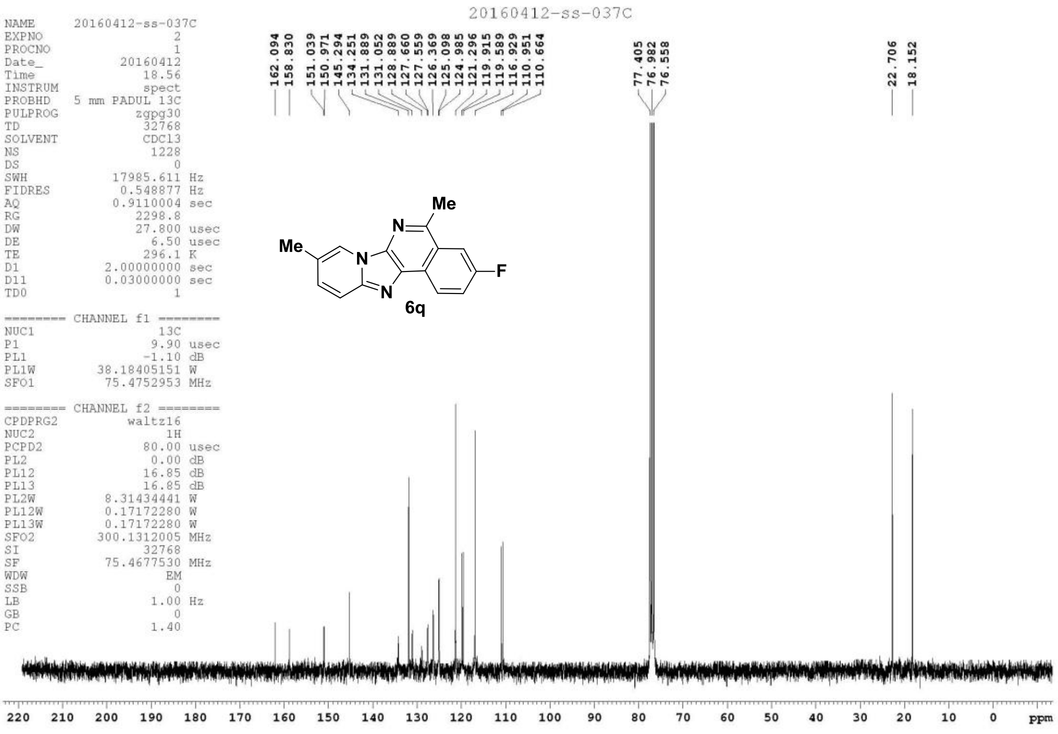
20160412-ss-037C
162.094
158.830
151.039
150.971
145.294
134.251
131.889
131.052
128.889
127.660
127.559
126.369
125.098
124.985
121.296
119.915
119.589
116.929
110.951
110.664
77.405
76.982
76.558
22.706
18.152
NAME 20160412-ss-037C
EXPNO 2
PROCNO 1
Date_ 20160412
Time 18.56
INSTRUM spect
PROBHD 5 mm PADUL 13C
PULPROG zgpg30
TD 32768
SOLVENT CDCl3
NS 1228
DS 0
SWH 17985.611 Hz
FIDRES 0.548877 Hz
AQ 0.9110004 sec
RG 2298.8
DW 27.800 usec
DE 6.50 usec
TE 296.1 K
D1 2.00000000 sec
D11 0.03000000 sec
TD0 1
======== CHANNEL f1 ========
NUC1 13C
P1 9.90 usec
PL1 -1.10 dB
PL1W 38.18405151 W
SFO1 75.4752953 MHz
======== CHANNEL f2 ========
CPDPRG2 waltz16
NUC2 1H
PCPD2 80.00 usec
PL2 0.00 dB
PL12 16.85 dB
PL13 16.85 dB
PL2W 8.31434441 W
PL12W 0.17172280 W
PL13W 0.17172280 W
SFO2 300.1312005 MHz
SI 32768
SF 75.4677530 MHz
WDW EM
SSB 0
LB 1.00 Hz
GB 0
PC 1.40
Me
N
Me
N
N
F
6q
220
210
200
190
180
170
160
150
140
130
120
110
100
90
80
70
60
50
40
30
20
10
0
ppm

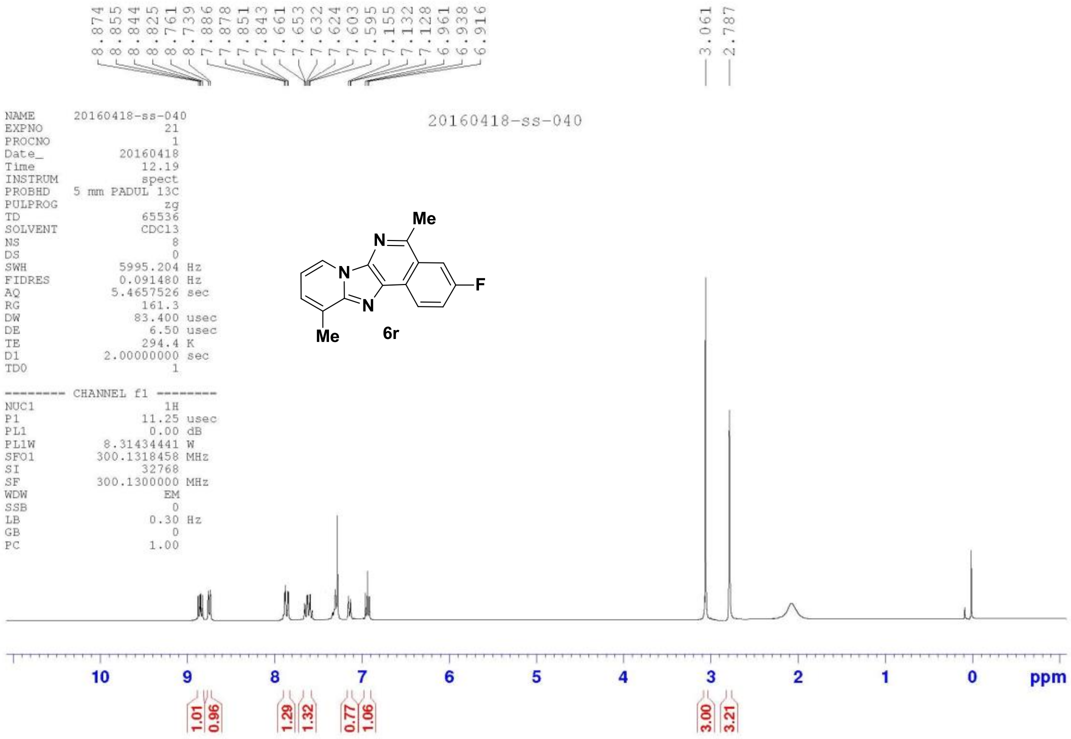
8.874 8.855 8.844 8.825 8.761 8.739 7.886 7.878 7.851 7.843 7.661 7.653 7.632 7.624 7.603 7.595 7.155 7.132 7.128 6.961 6.938 6.916
3.061 2.787
20160418-ss-040
NAME 20160418-ss-040
EXPNO 21
PROCNO 1
Date_ 20160418
Time 12.19
INSTRUM spect
PROBHD 5 mm PADUL 13C
PULPROG zg
TD 65536
SOLVENT CDCl3
NS 8
DS 0
SWH 5995.204 Hz
FIDRES 0.091480 Hz
AQ 5.4657526 sec
RG 161.3
DW 83.400 usec
DE 6.50 usec
TE 294.4 K
D1 2.00000000 sec
TD0 1
======== CHANNEL f1 ========
NUC1 1H
P1 11.25 usec
PL1 0.00 dB
PL1W 8.31434441 W
SFO1 300.1318458 MHz
SI 32768
SF 300.1300000 MHz
WDW EM
SSB 0
LB 0.30 Hz
GB 0
PC 1.00
Me
N
N
N
F
Me
6r
10 9 8 7 6 5 4 3 2 1 0 ppm
1.01 0.96 1.29 1.32 0.77 1.06 3.00 3.21

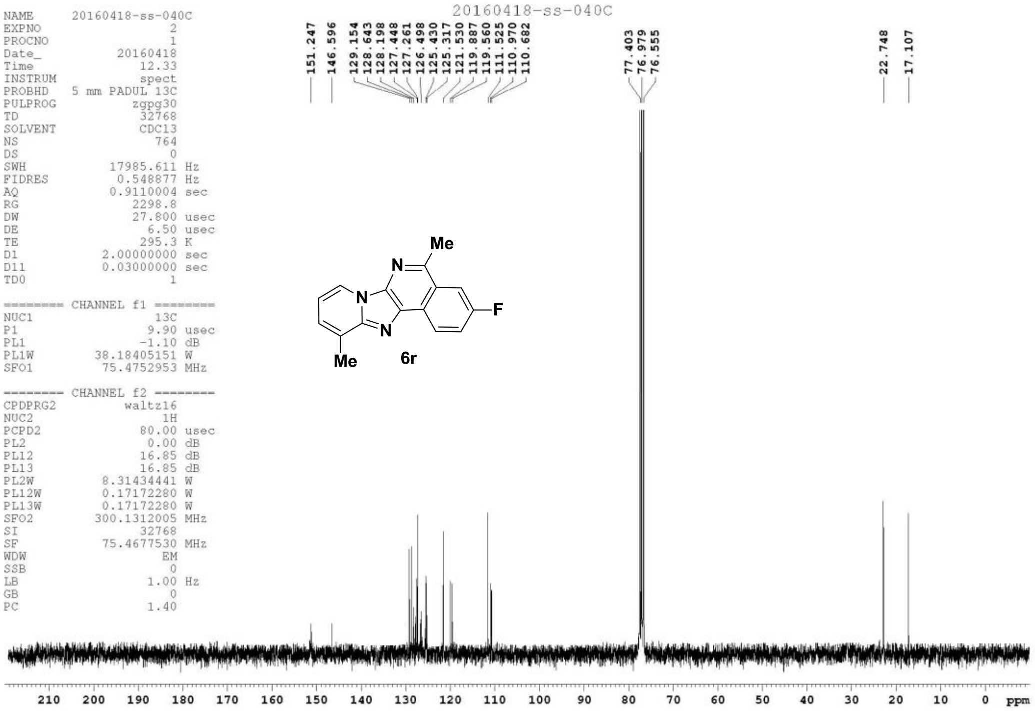
20160418-ss-040C
151.247 146.596 129.154 128.643 128.198 127.448 127.261 126.498 125.430 125.317 121.530 119.887 119.560 111.525 110.970 110.682
77.403 76.979 76.555
22.748 17.107
NAME 20160418-ss-040C
EXPNO 2
PROCNO 1
Date_ 20160418
Time 12.33
INSTRUM spect
PROBHD 5 mm PADUL 13C
PULPROG zgpg30
TD 32768
SOLVENT CDCl3
NS 764
DS 0
SWH 17985.611 Hz
FIDRES 0.548877 Hz
AQ 0.9110004 sec
RG 2298.8
DW 27.800 usec
DE 6.50 usec
TE 295.3 K
D1 2.00000000 sec
D11 0.03000000 sec
TD0 1
======== CHANNEL f1 ========
NUC1 13C
P1 9.90 usec
PL1 -1.10 dB
PL1W 38.18405151 W
SFO1 75.4752953 MHz
======== CHANNEL f2 ========
CPDPRG2 waltz16
NUC2 1H
PCPD2 80.00 usec
PL2 0.00 dB
PL12 16.85 dB
PL13 16.85 dB
PL2W 8.31434441 W
PL12W 0.17172280 W
PL13W 0.17172280 W
SFO2 300.1312005 MHz
SI 32768
SF 75.4677530 MHz
WDW EM
SSB 0
LB 1.00 Hz
GB 0
PC 1.40
Me
N
N
N
F
Me
6r
210 200 190 180 170 160 150 140 130 120 110 100 90 80 70 60 50 40 30 20 10 0 ppm

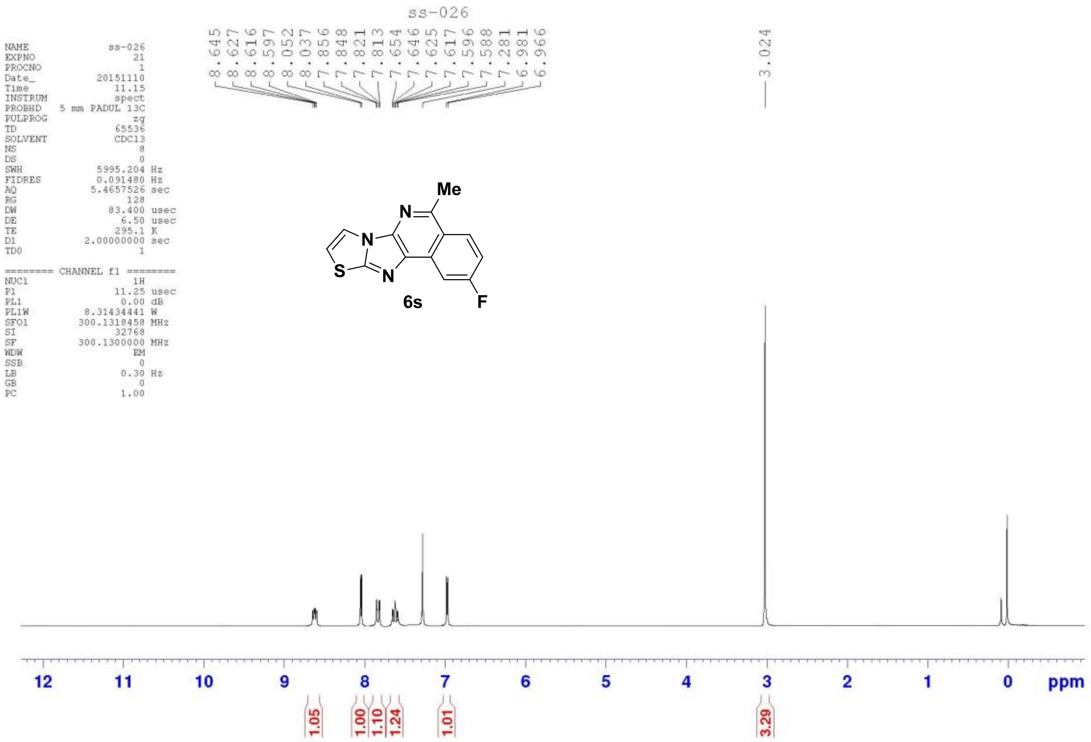
ss-026
8.645
8.627
8.616
8.597
8.052
8.037
7.856
7.848
7.821
7.813
7.654
7.646
7.625
7.617
7.596
7.588
7.281
6.981
6.966
3.024
NAME ss-026
EXPNO 21
PROCNO 1
Date_ 20151110
Time 11.15
INSTRUM spect
PROBHD 5 mm PADUL 13C
PULPROG zg
TD 65536
SOLVENT CDCl3
NS 8
DS 0
SWH 5995.204 Hz
FIDRES 0.091480 Hz
AQ 5.4657526 sec
RG 128
DW 83.400 usec
DE 6.50 usec
TE 295.1 K
D1 2.00000000 sec
TD0 1
======== CHANNEL f1 ========
NUC1 1H
P1 11.25 usec
PL1 0.00 dB
PL1W 8.31434441 W
SFO1 300.1318458 MHz
SI 32768
SF 300.1300000 MHz
WDW EM
SSB 0
LB 0.30 Hz
GB 0
PC 1.00
Me
N
N
S
N
F
6s
12
11
10
9
8
7
6
5
4
3
2
1
0
ppm
1.05
1.00
1.10
1.24
1.01
3.29

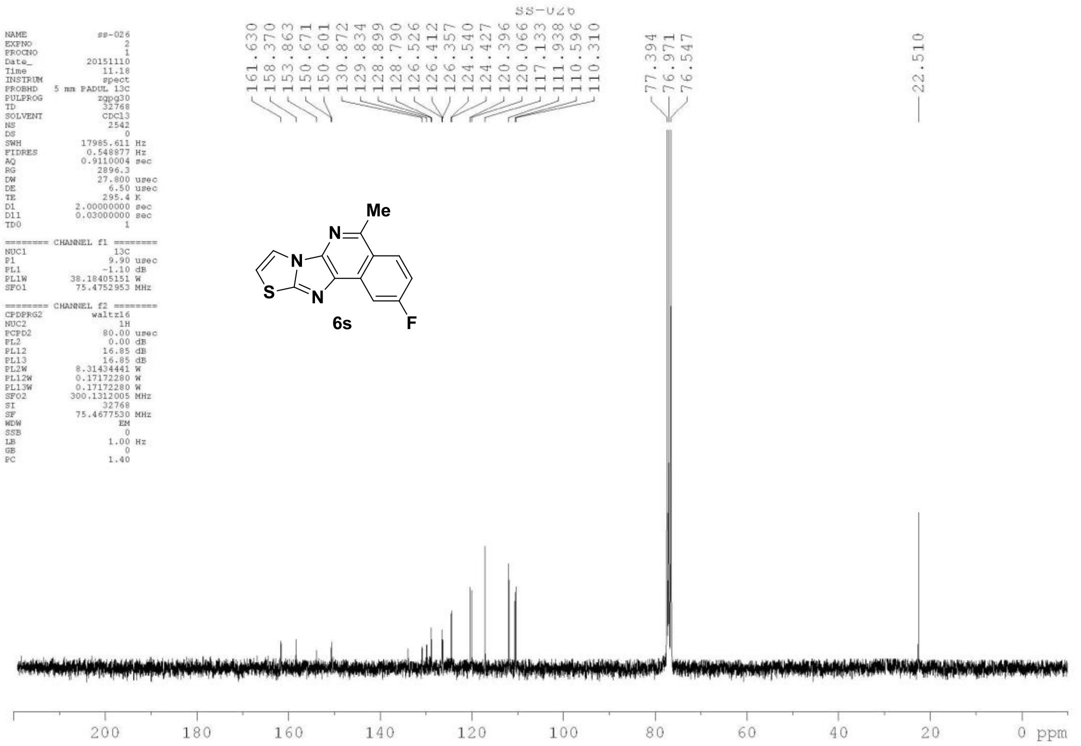
ss-026
161.630
158.370
153.863
150.671
150.601
130.872
129.834
128.899
128.790
126.526
126.412
126.357
124.540
124.427
120.396
120.066
117.133
111.938
110.596
110.310
77.394
76.971
76.547
22.510
NAME ss-026
EXPNO 2
PROCNO 1
Date_ 20151110
Time 11.18
INSTRUM spect
PROBHD 5 mm PADUL 13C
PULPROG zgpg30
TD 32768
SOLVENT CDCl3
NS 2542
DS 0
SWH 17985.611 Hz
FIDRES 0.548877 Hz
AQ 0.9110004 sec
RG 2896.3
DW 27.800 usec
DE 6.50 usec
TE 295.4 K
D1 2.00000000 sec
D11 0.03000000 sec
TD0 1
======== CHANNEL f1 ========
NUC1 13C
P1 9.90 usec
PL1 -1.10 dB
PL1W 38.18405151 W
SFO1 75.4752953 MHz
======== CHANNEL f2 ========
CPDPRG2 waltz16
NUC2 1H
PCPD2 80.00 usec
PL2 0.00 dB
PL12 16.85 dB
PL13 16.85 dB
PL2W 8.31434441 W
PL12W 0.17172280 W
PL13W 0.17172280 W
SFO2 300.1312005 MHz
SI 32768
SF 75.4677530 MHz
WDW EM
SSB 0
LB 1.00 Hz
GB 0
PC 1.40
Me
N
N
S
N
F
6s
200
180
160
140
120
100
80
60
40
20
0 ppm
